# Effects of ciprofloxacin on bacterial abundance and enrichments in samples taken from the sea surface microlayer and underlying waters in the southern North Sea

**DOI:** 10.3389/fmicb.2025.1624041

**Published:** 2025-08-27

**Authors:** Adenike Adenaya, Florence Zumbika, Ruben Rios-Quintero, Pablo A. Lara-Martín, Oliver Wurl, Martin Könneke, Mariana Ribas-Ribas, Axel Hamprecht, Thorsten Brinkhoff

**Affiliations:** ^1^Institute for Chemistry and Biology of the Marine Environment (ICBM), School of Mathematics and Science, Carl von Ossietzky University of Oldenburg, Oldenburg, Germany; ^2^Physical Chemistry Department, Faculty of Marine and Environmental Sciences, University of Cádiz, CEI·MAR, Cádiz, Spain; ^3^Center for Marine Sensors (ZfMarS), Institute for Chemistry and Biology of the Marine Environment (ICBM), School of Mathematics and Science, Carl von Ossietzky University of Oldenburg, Oldenburg, Germany; ^4^University Institute of Medical Microbiology and Virology, University of Oldenburg, Oldenburg, Germany

**Keywords:** sea surface microlayer, coastal waters, antibiotic-resistant bacteria, antibiotics, marine bacteria, southern North Sea, underlying waters

## Abstract

The sea surface microlayer (SML), a biofilm-like environment, is a unique and challenging habitat for microbes, due to its position at the interface between the ocean and the atmosphere. In coastal areas, microbial communities in the SML are often exposed to anthropogenic pollutants, including heavy metals, microplastics, and pharmaceuticals. While studies have been conducted on the effects of some of these pollutants, further research is needed to understand the impact of antibiotics on the development of bacterial resistance in the SML. Ciprofloxacin showed high effectiveness against SML bacteria in a previous study. Thus, we investigated the effects of different concentrations of this antibiotic on the dynamics of bacterial communities in samples of the SML and the corresponding underlying water (ULW) over time. Ciprofloxacin concentrations of 50 and 100 ng/mL affected bacterial cell numbers and exerted selective pressure on bacterial communities. A non-metric multidimensional scaling (dissimilarity matrix) revealed significant differences in the bacterial community compositions at different time points, regardless of the ciprofloxacin concentration, and indicated that the combination of time and ciprofloxacin concentrations impacts bacterial communities in the SML and ULW (*r*^2^ = 67%, *p* = 0.001). Marine bacteria of the *Rhodobacterales*, including the genera *Planktomarina, Lentibacter,* and unknown *Rhodobacteraceae,* persist in the presence of 100 ng/mL ciprofloxacin over time. The abundance of *Campylobacterales*, particularly *Arcobacteraceae,* increased over time and with increasing ciprofloxacin concentrations, raising concerns about the development and spread of potential antibiotic-resistant pathogens in the SML and ULW. Ninety-seven bacterial strains (42 marine, 55 non-marine) belonging to 14 genera were also enriched and isolated in the presence of 100 ng/mL ciprofloxacin. Further antibiotic susceptibility tests on enriched marine bacteria revealed widespread resistance to ciprofloxacin and other antibiotics found in environmental samples. Our study, therefore, suggests that more efforts are needed to safeguard the integrity of coastal environments and to mitigate the spread of antibiotic-resistant bacteria in the ecosystem.

## 1 Introduction

Antibiotics were discovered nine decades ago to treat infectious diseases and reduce human mortality (Hutchings et al., 2019). However, the rise in global consumption of these drugs has escalated the antibiotic resistance of bacteria and diminished options for infectious disease treatments (Spellberg and Gilbert, 2014). Cohen (2000) predicted antibiotic resistance as a major public health issue in the 21st century, possibly paving the way for pandemics. Thus, despite the success of antibiotics in infectious disease prevention and control, antibiotic resistance could result in about 10 million deaths globally in 2050 (O’Neill, 2016). In 2017, global antibiotic consumption reached 93 million tons, with projections indicating that it will surpass 236 million by 2030 (Tiseo et al., 2020). Several frameworks and policies have been developed to reduce and mitigate the spread of antibiotic-resistant bacteria. An example is the One Health framework that was developed by the World Health Organization (WHO, 2024) to monitor and control the spread of infectious diseases and antibiotic-resistant bacteria between humans, animals, and the environment.

Ciprofloxacin is a broad-spectrum antibiotic belonging to the fluoroquinolone class, used in both clinical and aquacultural settings to treat diseases in humans and fish (Rehman et al., 2019; Wang et al., 2021). The antibiotic works by inhibiting bacterial replication, making it an effective bactericidal agent. However, ciprofloxacin is not fully metabolized by the human body, leading to the excretion of 20 to 60% of the antibiotic into municipal wastewater (Al-Omar, 2005). As a result, concentrations of ciprofloxacin in wastewater influents and effluents can reach up to 2,831 ng/L and 564 ng/L, respectively (Biel-Maeso et al., 2018). In densely populated and highly industrialized regions, the concentration of ciprofloxacin in wastewater effluents can be as high as 14 mg/L (Fick et al., 2009). Due to the continuous discharge of wastewater effluents into coastal waters (Biel-Maeso et al., 2018; Fick et al., 2009), there are concerns about contaminants, such as antibiotics, and their effects on marine bacteria (Haenni et al., 2022).

The presence of antibiotics in coastal water poses a significant threat to the bacterial communities. Of particular interest are the effects on the bacterial communities in the ubiquitous sea surface microlayer (SML), which is typically less than 1 mm thick, and the underlying water (ULW), which is typically mixed. This focus is important due to the existing knowledge gap in understanding the effects of antibiotics on the bacterial dynamics and interaction in these environments. The SML is an essential fraction of the ocean with observed biofilms, allowing for high bacterial proliferation (Wurl et al., 2016). The bacterial communities in the SML are ecologically crucial for the remineralization of organic matter and nutrient recycling (Reinthaler et al., 2008; Wurl et al., 2016). Nonetheless, the SML presents unique challenges to bacterial communities due to its position between the ocean and the atmosphere. Bacterial communities are continuously exposed to environmental factors such as temperature and salinity fluctuations, ultraviolet radiation, and wind speed at the interface (Stolle et al., 2011). Thus, contaminants such as antibiotics in the SML could further contribute to the ecological stressors faced by bacterial communities.

Recently, we proposed that the SML of an anthropogenically influenced coastal environment could act as a hotspot for antibiotic-resistant bacteria (Adenaya et al., 2023a). This hypothesis was supported by a previous study, which found that bacteria isolated from the SML in the Jade Bay in the southern North Sea exhibited resistance to low concentrations of antibiotics (Adenaya et al., 2024a). Furthermore, genome analysis of three antibiotic-resistant bacteria belonging to the *Pseudoseohaeicola*, *Nereida*, and *Vibrio* genera, isolated from the SML, revealed the presence of high numbers of antibiotic resistance genes, highlighting their adaptation to an already challenging habitat (Adenaya et al., 2024a). Among the 16 antibiotics tested, ciprofloxacin proved to be one of the most effective. Considering that the antibiotic is widely used in both clinical and aquacultural settings (Al-Omar, 2005; Berntssen et al., 2014) as well as its prevalence in coastal waters (Biel-Maeso et al., 2018), we chose this antibiotic to investigate its impact on the abundance and enrichment of resistant bacteria in environmental SML and ULW samples collected from Jade Bay. Our study also aimed to assess the presence of antibiotics in the environmental SML and ULW samples. Additionally, we aim to examine the resistance of enriched and isolated bacteria to the antibiotics that may be present in the environment. Our study, therefore, showed that the influx of antibiotics into coastal waters may facilitate the development and spread of antibiotic-resistant bacteria within the SML and the ULW.

## 2 Materials and methods

### 2.1 Sampling site and sample collections

**Figure 1:**
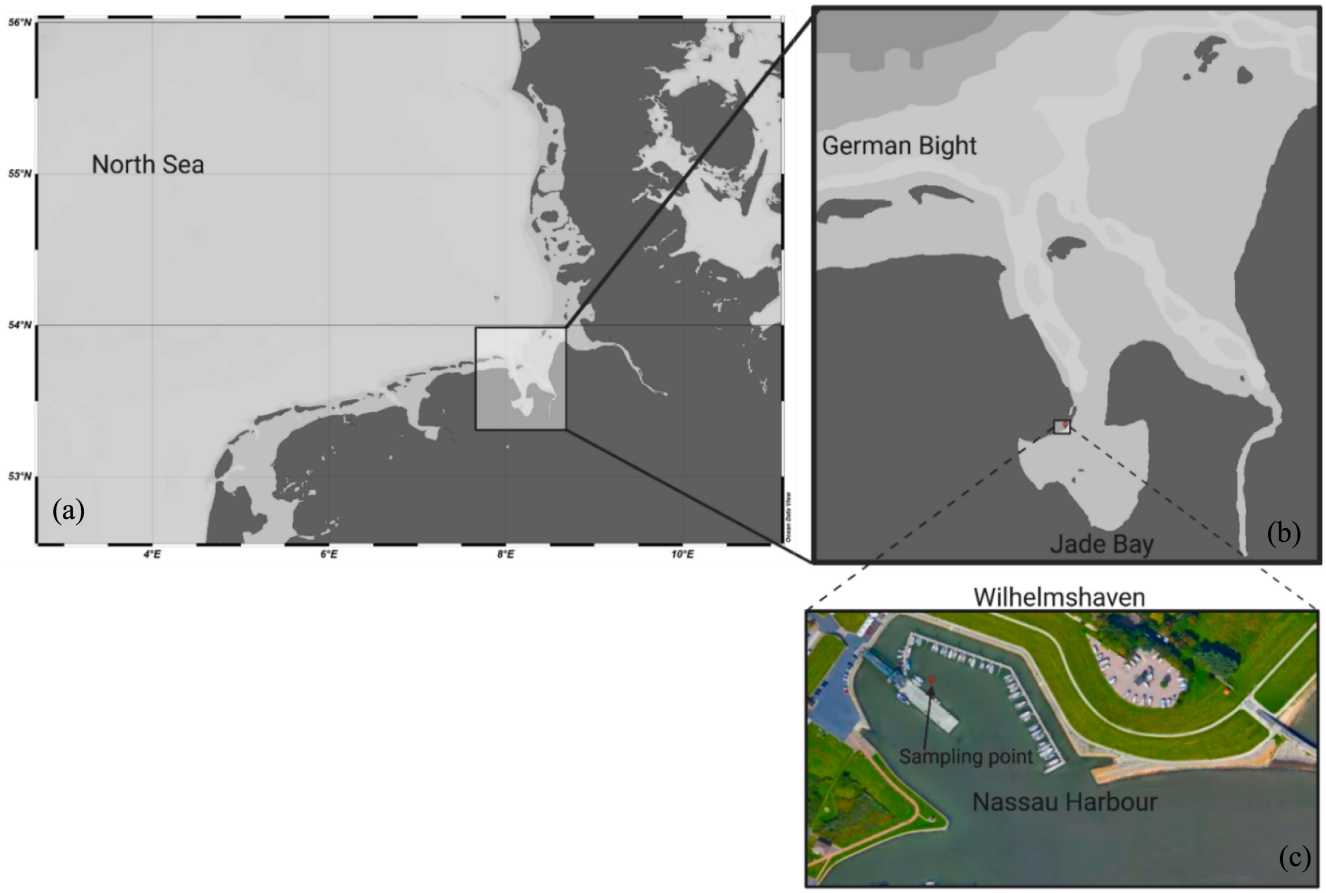
**(a,b)** Map showing the North Sea and the Jade Bay. **(c)** Enlargement of the Nassau Harbor in the city of Wilhelmshaven. The arrow points to the spot where SML and ULW samples were taken. The map was generated using Ocean Data View (https://odv.awi.de, 2023) and Google Maps (https://www.google.com/maps).

Seawater samples from the SML and the ULW were collected on March 17, 2023, in the Nassau Harbor of Wilhelmshaven, located at the Jade Bay in the southern North Sea (Figure 1). The Jade Bay is an enclosed coastal area, strongly exposed to various anthropogenic activities, including wastewater treatment plants (Adenaya et al., 2024b) and sometimes chemical and oil spills (Kreutzer et al., 2023). SML samples were collected using the glass plate method described by Harvey and Burzell (1972). Before sampling, the glass plate was cleaned with high-grade ethanol (90%) and then rinsed by dipping it into seawater. For microbiological analysis, the SML sample that adhered to the glass plate was wiped with a sterile squeegee and then transferred into sterile 1 L bottles, which had been pre-rinsed with the SML. A sample volume of 4 L was collected. ULW samples from 1 m depth were collected using sterile syringes connected to a sterile hose and into sterile bottles. Furthermore, we collected 500 mL of SML and ULW (in duplicate) for chemical analysis into amber high-density polyethylene bottles, rinsing them with high-grade ethanol, high-quality MQ water, and the SML/ULW sample.

Environmental parameters, such as temperature and conductivity, were measured using a CyberScan PCD 650-meter probe (Oakton USA) at the sea surface and at a depth of 1 m. The temperatures of the air, seawater surface, and at a depth of 1 m were 10°C, 6°C, and 4°C, respectively. The salinity of the seawater was ∼28 g/kg, and the pH was 7.8. All samples were maintained on ice, transported to the laboratory within 2 h of collection, and processed immediately. The SML and ULW samples were pooled and homogenized in sterile 4 L bottles and then processed for subsequent analysis. Samples collected for analytical purposes were stored at −80°C in the dark until they were analyzed.

### 2.2 Determination of bacterial cell counts in samples treated with ciprofloxacin

To assess the effects of ciprofloxacin on bacterial communities in environmental samples, 300 mL of homogenized SML/ULW samples were treated with 0, 10, 50, and 100 ng/mL ciprofloxacin in 500 mL sterile flasks (in triplicate). While these concentrations may not be typical in coastal waters, they are clinically relevant and have been used to examine changes in bacterial communities in pristine aquatic environments (Marti et al., 2016). Samples were incubated at 4°C (the same temperature as measured in the environmental sample) on a shaker in the dark. Aliquots of 1 mL were taken from the flasks at three different time points (T0, T1, and T2) to determine bacterial cell counts via flow cytometry. T0 samples were collected immediately after adding the antibiotic, while T1 and T2 samples were collected after three and 7 days of incubation, respectively. Aliquots were fixed with 1% glutaraldehyde and further processed as previously described by Giebel et al. (2019). In brief, samples were diluted 1:10 with phosphate-buffered saline (PBS) solution, and 400 μL of each sample was stained with 20 μL of SYBR Green I Nucleic Acid Gel Stain (9 × in final concentration, Molecular Probes, Invitrogen, Germany). The mixture was incubated in the dark at room temperature for 30 min. Cell numbers were then measured using an Accuri C6-1 Flow Cytometer (BD Bioscience, United States). The enrichment factor (EF) of bacteria at different concentrations of ciprofloxacin was calculated by dividing the cell numbers in the SML by those in the ULW at different time points. EF is the ratio of biological and non-biological entities measured in the SML to those measured in the ULW (Rahlff et al., 2017). EF typically ≥ 1 indicates higher enrichment of bacteria in the SML than in the ULW, while an EF < 1 indicates otherwise (Rahlff et al., 2017).

### 2.3 Enrichment and isolation of bacteria

To enrich resistant bacteria in the presence of ciprofloxacin, 0.5 mL of each SML and ULW sample was inoculated into 4.5 mL of marine broth containing 0, 10, 50, and 100 ng/mL of ciprofloxacin. Samples were incubated at 20°C on a shaker in the dark, and growth was determined by measuring the optical density (OD_600_) for 7 days. At the early exponential phase, i.e., day 2, 10 μL from the enrichment cultures with different ciprofloxacin concentrations were transferred onto marine agar (MA) and Mueller-Hinton agar (MHA) amended with the same ciprofloxacin concentration. Isolates obtained on MA were incubated at 20°C, and those obtained on MHA were incubated at 37°C in the dark for 72 h. MA enhances the growth of marine bacteria (Rodrigues and de Carvalho, 2022), which grow best between 15°C and 25°C (e.g., Hahnke et al., 2024) while MHA facilitates the growth of non-marine bacteria, including pathogens that grow between 35 and 37°C. After observing that bacteria also grew at the highest ciprofloxacin concentration, we selected only colonies that grew with the highest concentration, i.e., 100 ng/mL, as representatives of ciprofloxacin-resistant bacteria for further analysis. These colonies were subsequently and continuously transferred onto plates with 100 ng/mL of ciprofloxacin until pure colonies were achieved. All pure isolates were stored as glycerol stocks (50%) at −80°C.

### 2.4 Identification of enriched bacterial isolates

For identification of the isolates cultivated on MA, we carried out a freeze-and-thaw method to extract their DNA and amplified their 16S rRNA genes using the primer set 27f; 5′- AGA GTT TGA TCM TGG CTC AG-3′ and 1492r; TAC GGY TAC CTT GTT ACG ACT T-3′ (Brinkhoff and Muyzer, 1997). PCR products were analyzed on 1% (w/v) agarose gels (Sigma-Aldrich, Germany) in 1% TAE. Purification was carried out using the QIAquick PCR purification kit (QIAGEN, Germany). Sequencing was carried out by the GATC sequencing company (Eurofins, Germany). Phylogenetic affiliations were determined using the NCBI’s Basic Local Alignment Search Tool (BLAST; http://ncbi.nlm.nih.gov). Based on their 16S rRNA gene sequences, the phylogenetic tree of bacteria was constructed using the Geneious software (version 2025.0). The tree was constructed using the neighbor-joining method and the Tamura-Nei genetic distance model after multiple alignments. The bootstrapping iteration was set to 100 times. The 16S rRNA gene sequences of cultivated isolates were deposited in NCBI GenBank under the accession numbers OR739618-OR739687. Bacteria grown on MHA were identified using the Matrix-Assisted Laser Desorption/Ionization Time-of-Flight (MALDI-TOF) (Bruker Daltonics, Germany) according to the protocol established by Hamprecht et al. (2014). The 16S rRNA genes of bacterial isolates not in the MALDI-TOF database or having a MALDI-TOF score < 2.0 were sequenced as described above.

### 2.5 Community analysis via 16S rRNA amplicon sequencing

A total of 99 mL of SML/ULW samples, treated with 0, 10, 50, and 100 ng/mL ciprofloxacin, were collected from the flasks at each time point to analyze the bacterial community using 16S rRNA amplicon sequencing. The samples were filtered through 0.2 μm polycarbonate nucleopore filters (Sigma-Aldrich, Germany) and stored at −80°C until further analysis. The genomic DNA on the filters was extracted using the phenol-chloroform method as described by Brick et al. (2025). DNA quantification was assessed using a NanoDrop 1,000 (Thermo Fisher, United States). To ensure a uniform DNA quantity for PCR, the concentrations of extracted DNA were adjusted by dilution with PCR-grade water. Extracted DNA was amplified using the primers 515F-Y (5′-GTG YCA GCM GCC GCG GTA A-3′) and the 926R (5’-CCG YCA ATT YMT TTR AGT TT-3′) which specifically target the hypervariable V4–V5 regions of 16S rRNA genes (Parada et al., 2016). The PCR reaction consisted of 2 μL of target DNA, combined with 5 μL of 5x Phusion-HF buffer, 0.5 μL of dNTPs (200 μM), 1.25 μL of each primer (0.5 μM), 0.25 μL of Phusion-Taq polymerase (1 U/50 μL), and 14.75 μL of PCR-grade water, resulting in a total reaction volume of 25 μL per sample. PCR was performed on a Mastercycler Nexus X2 (Eppendorf, Germany). PCR products were analyzed using 1% (w/v) agarose gels (Sigma-Aldrich, Germany) in 1% TAE. Biological triplicates were pooled, and the resultant PCR product was purified using AMPure XP beads (Beckman Coulter, United States). Quantification of the purified PCR products was performed using Qubit (Thermo Fisher, United States), and sequencing was carried out using an in-house Illumina NextSeq 1,000 sequencer (Illumina, Germany).

### 2.6 Bioinformatic processing of amplicon data

Bioinformatic processing of amplicon data was performed using an in-house pipeline, as fully described by Milke et al. (2022) and Brick et al. (2025). In brief, the raw reads were quality filtered with cutadapt and trimmed to 210 bp for both forward and reverse reads to eliminate low-quality ends. Sequence denoising, merging, and removal of chimeric reads were carried out using the DADA2 algorithm pipeline through the QIIME2 plugin q2-dada2. The QIIME2 classify-sklearn plugin was then used to conduct a sequence similarity search against the SILVA138 database, enabling the clustering of sequences into amplicon sequence variants (ASVs). ASVs were converted into relative abundance with a focus on the order and genus of bacterial taxonomic units. Sequences of the 18S rRNA gene were excluded from the dataset, and the subsequent 16S rRNA sequence reads generated were deposited in the European Nucleotide Archive under the project accession number PRJEB91248.

### 2.7 Chemical analysis of antibiotics in environmental SML and ULW samples

Information on all target antibiotics is provided in Supplementary Table S1. Solvents used for the chemical analysis of target antibiotics were of high chromatography standards, as recently described by Adenaya et al. (2024a). Methanol and water (HPLC and LC–MS grades) were purchased from Scharlau (Spain), and the target antibiotics were obtained from Sigma-Aldrich (Spain). Milli-Q water and high-quality ethanol were used for all cleaning procedures to reduce matrix effects. Sample preparation, extraction, chromatography conditions, calibration, and cleaning procedures were all carried out using the protocol described by Baena-Nogueras et al. (2016) and Adenaya et al. (2024a). Briefly, we used the solid phase extraction (SPE) method with the SPE instrument (Dionex™, AutoTrace™ 280, Thermo Fisher, USA) and 200 mg Oasis HLB cartridges (Waters, United States) to extract target antibiotic compounds. Prior to this, each sample pH was adjusted to 2 for maximum recovery (Baena-Nogueras et al., 2016). 50 μL of isotopically labeled surrogate compounds (ibuprofen-D_3_ and carbamazepine-D_10_) was added to each sample to verify the extraction method (Baena-Nogueras et al., 2016). Each sample was passed through the SPE columns at a flow rate of 4 mL/min. The extracted sorbent was washed twice with 10 mL of HPLC-grade water and air-dried for 10 min. The extracts were eluted into clean tubes using LC–MS-grade methanol and evaporated under nitrogen flow for 1 h. The extracts were later resuspended in a 50:50 (v/v) mixture of methanol and water, sonicated for 5 min at room temperature, and then filtered through 0.22 μm polytetrafluoroethylene filters (Teknokroma, Spain). Chemical analysis of target antibiotics in the SML and ULW samples was carried out by ultra-performance liquid chromatography-triple quadrupole mass spectrometry in tandem with ionization spray (UPLC-QqQMS/MS, Bruker, United States).

The C18 Intensity Solo UPLC Column (2.1 mm x 100 mm, 1.8 μm pore size; Acquity, Waters, Ireland) was used for the chromatographic separations of antibiotics. Antibiotics analyzed in this study were obtained under positive and negative electrospray modes (ESI+/−). In both cases, the aqueous phase consisted of water, and the organic phase was methanol (both LC–MS grade). For ESI + antibiotics, both solutions were added with 5 mM of formic acid and 5 mM of ammonium formate (pH = 3). For ESI-antibiotics, with 5 mM ammonia and 5 mM ammonium acetate (pH = 8). The sample and standard injection volumes were 10 μL, and the total system run time per sample was 10 min, including a 2-min re-equilibration time. The elution gradient for both ESI modes started at 5% of methanol and 95% of water; the percentage of methanol increased to 100% over the first 3 min. The flow rate, pressure, and temperature were set at 0.4 mL/min, 0.4 bar, and 40°C, respectively. Samples were analyzed in duplicates. Target antibiotics were identified in the samples by matching their retention times and signal intensities to those of the standards. The multiple reaction monitoring (MRM), retention time, limit of detection (LOD), and limit of quantification (LOQ) of target antibiotics were obtained from Adenaya et al. (2024a).

### 2.8 Antibiotic susceptibility tests

All antibiotic disks were purchased from Oxoid (United Kingdom). Antibiotic susceptibility tests were conducted for marine bacteria enriched in the SML and ULW samples and isolated on marine agar continuously treated with 100 ng/mL of ciprofloxacin. Antibiotic susceptibility tests were conducted to further evaluate the resistance of enriched and cultivated bacterial isolates to ciprofloxacin (CIP; 5 μg). Bacterial isolates were also subjected to novobiocin (NV; 5 μg), ofloxacin (OFL; 5 μg), clarithromycin (CLR; 15 μg), erythromycin (E; 10 μg), and clindamycin (DA; 10 μg). Although other concentration units, such as ng/mL, were used for bacterial cell counts, enrichments, and community analysis, the antibiotics in this section are expressed in μg, following the standardized disk diffusion method of antibiotic susceptibility testing. Marine bacteria lack a standardized method for antibiotic testing and interpretation. Therefore, we used a modified EUCAST (2024) guideline to accommodate the growth requirements and interpretation of inhibition zones. For the former, MA was used for antibiotic susceptibility tests. Fresh liquid cultures were prepared by inoculating a pure colony of each strain separately in 5 mL of marine broth. Incubation was carried out at 20°C on a shaker for 24 h. Bacterial growth was monitored by measuring the optical density (OD_600_), and the cultures were adjusted to an optical density of 0.5. A volume of 200 μL of each bacterial culture was spread on agar plates, and antibiotic disks were carefully placed on the plates. Incubation was performed for a maximum period of 24 h at 20°C, and zones of inhibition were measured in mm. For the interpretation of zones of inhibition, we used the EUCAST specified breakpoints of *Enterobacterales* for ciprofloxacin and ofloxacin (resistant if <22 mm). Of *Vibrio* spp. for clarithromycin and erythromycin (resistant if <12 mm), and of *Bacillus* spp. for clindamycin (resistant if <17 mm). Since novobiocin was not included in the EUCAST breakpoint guidelines, an arbitrary (Baker et al., 2016) breakpoint of < 16 mm was used to determine resistance.

### 2.9 Statistical analysis

Statistical analysis was conducted using R (version 4.3.3). The normality of the data pertaining to bacterial cell counts was assessed using the Shapiro–Wilk test, and paired *t*-tests were employed to investigate variations in bacterial cell numbers between SML and ULW samples across all concentrations and time points. To evaluate bacterial growth linearity at varying ciprofloxacin concentrations over 7 days, linear regression was utilized. Venn diagrams were created with Venny 2.1^1^ to analyze the proportions of individual and shared ASVs across different ciprofloxacin concentrations at various time points. A non-metric multidimensional scaling (NMDS) plot was constructed using the Bray–Curtis distance matrix (*K* = 2) in R (Vegan) to visualize community composition differences (Brick et al., 2025) across all concentrations and time points. The PERMANOVA test (with 999 permutations) was employed to statistically evaluate the response of bacterial communities in the SML and ULW samples to varying ciprofloxacin concentrations across different time points. The Shannon diversity index for bacterial communities in the SML and ULW samples was calculated using Vegan. Pairwise correlations (Spearman) were conducted to examine the correlation of the 20 most abundant bacterial taxa (genera) at the highest ciprofloxacin concentration (100 ng/mL) at T2. A significance threshold of *p* < 0.005 was set for all analyses, with a 95% confidence interval. Graphical representations were generated using the R (ggplot2) package.

## 3 Results

### 3.1 Bacterial cell counts in samples treated with different ciprofloxacin concentrations

**Figure 2:**
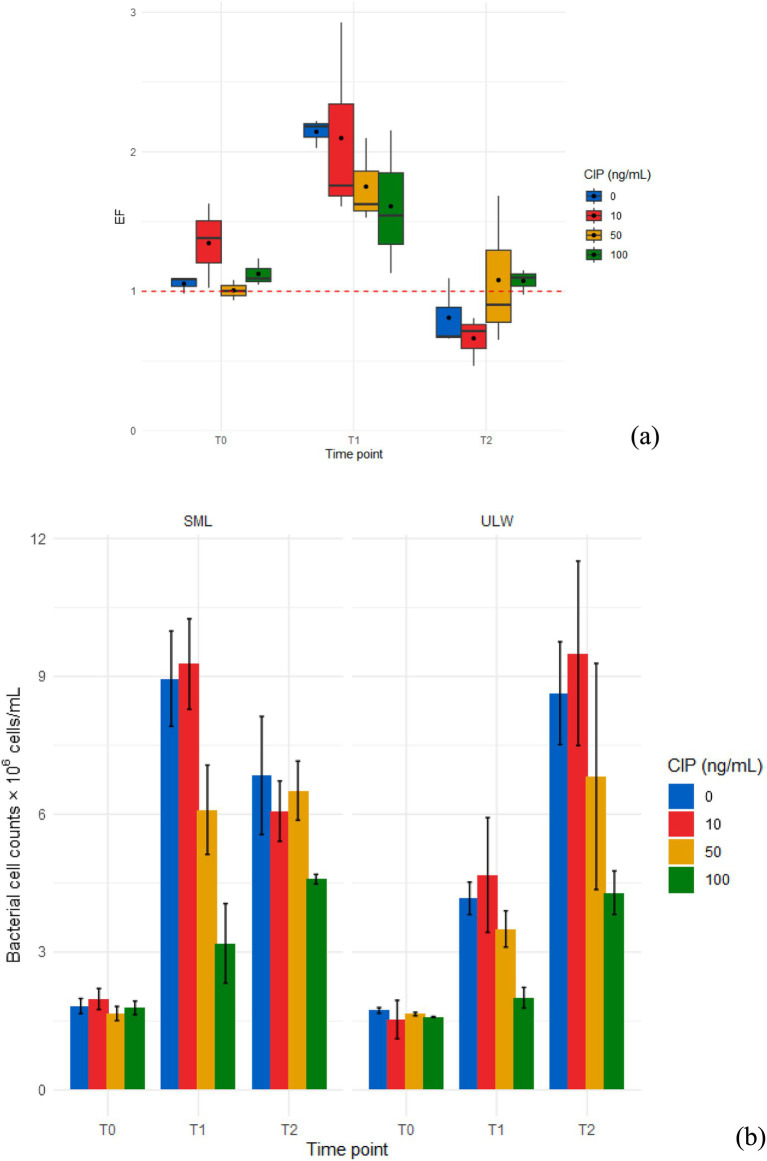
**(a)** Enrichment factor (EF) of bacteria in the SML and ULW samples treated with different ciprofloxacin (CIP) concentrations. **(b)** Bacterial cell counts in the SML and ULW across different ciprofloxacin concentrations and time points. T0 samples were taken on the sampling day, T1 samples after 3 days, and T2 samples after 7 days of incubation. Error bars show the standard deviations between three biological replicates.

The mean EF was >1, with a statistically significant difference (*p* < 0.001) observed in bacterial cell counts between the SML and the ULW samples at T1 (Figure 2a and Supplementary Figure S1). At T0 and T2, no significant differences were detected in bacterial counts between the SML and ULW. However, the ULW samples exhibited a higher overall count of bacterial cells at T2. Bacterial cell counts in both environments exhibited similar trends across different ciprofloxacin concentrations and time points. At T0, cell counts were consistent, ranging from 1.7 × 10^6^ ± 0.1 × 10^6^ cells/mL to 2.0 × 10^6^ ± 0.2 × 10^6^ cells/mL in the SML, and from 1.5 × 10^6^ ± 3.8 × 10^6^ cells/mL to 1.7 × 10^6^ ± 0.1 × 10^6^ cells/mL in the ULW, regardless of the ciprofloxacin concentration (Figure 2b). Subsequently, cell counts in SML samples increased significantly at T1, with more cells observed in the 0 and 10 ng/mL concentration than in the 50 and 100 ng/mL concentration (*p* < 0.001). Cell counts decreased at T2, with no significant difference observed across all concentrations. In ULW samples, cell counts increased across all concentrations at T1. However, at T2, cell counts increased significantly, with a notable difference between lower ciprofloxacin concentrations, i.e., 0 and 10 ng/mL, and 100 ng/mL (*p* < 0.001). Overall, ciprofloxacin concentrations of 50 ng/mL and 100 ng/mL affected bacterial cell counts in both the SML and ULW samples, resulting in lower cell counts compared to samples with lower ciprofloxacin concentrations. Nevertheless, cell counts in these concentrations increased with time, i.e., from T0 to T1 to T2, despite the lack of added nutrients to support bacterial growth.

### 3.2 Bacterial growth in enrichment cultures in different concentrations

**Figure 3:**
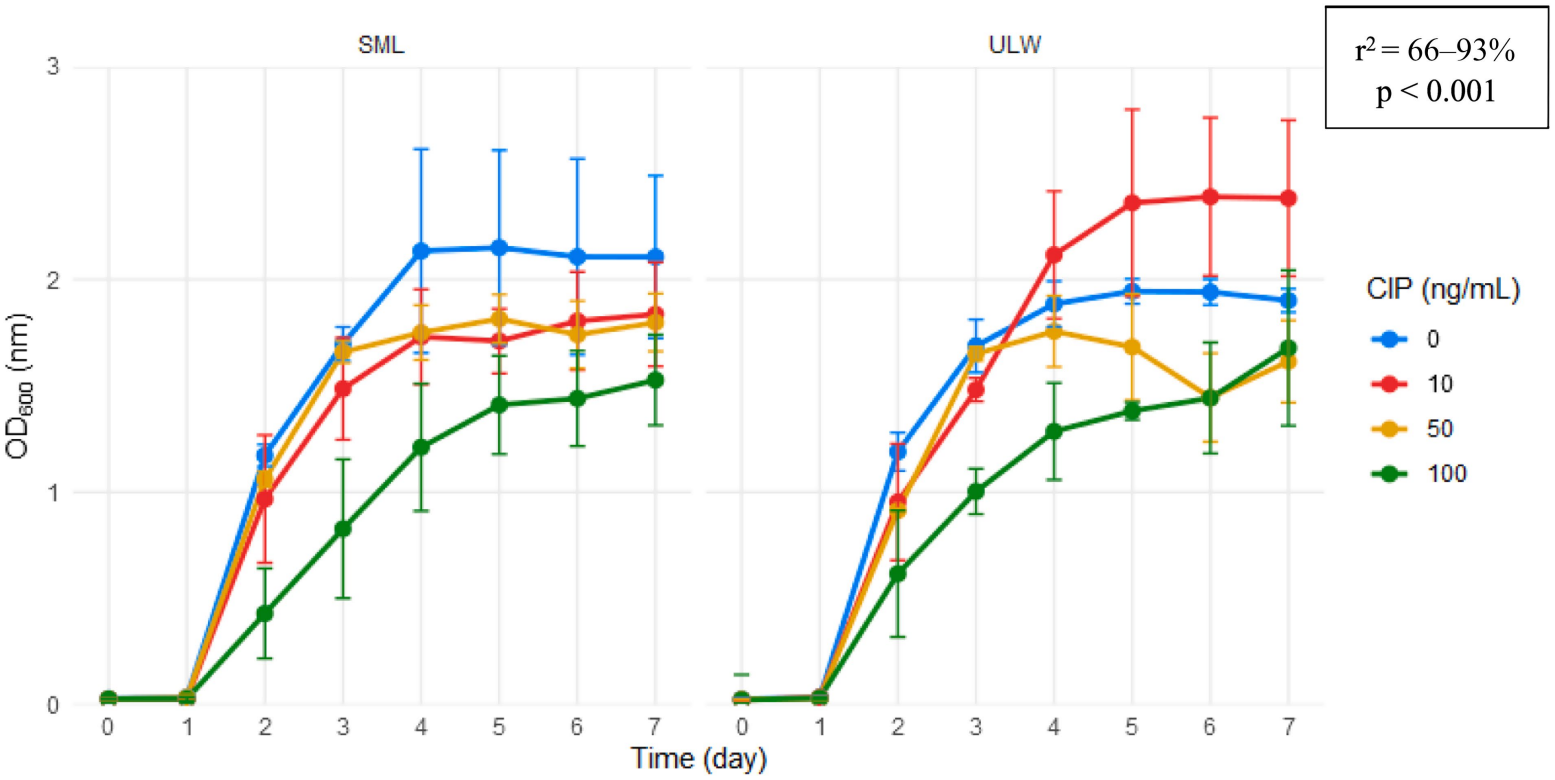
Growth (OD_600_) of SML and ULW bacterial cells in enrichment cultures in marine broth treated with different ciprofloxacin (CIP) concentrations. Error bars indicate the standard deviation of three biological replicates.

The trends observed in the enrichment culture in marine broth reflected the patterns of bacterial cell counts across all ciprofloxacin concentrations. Linear regression analysis revealed that bacterial growth occurred linearly, regardless of ciprofloxacin concentration (Figure 3). In the SML, more growth occurred in samples without ciprofloxacin, and in the ULW, more growth occurred in samples treated with 10 ng/mL. At concentrations of 50 and 100 ng/mL, ciprofloxacin had an impact on bacterial growth; however, growth at these concentrations also increased significantly over time. Indeed, bacterial growth in 100 ng/mL never reached a peak throughout the experiment and continued linearly with linear regressions of *r*^2^ > 90%, *p* < 0.001 in both the SML and ULW.

### 3.3 Identified bacterial isolates in the highest concentration

In total, we retrieved 97 bacterial isolates belonging to 14 different genera and able to grow in the presence of 100 ng/mL of ciprofloxacin. As expected, the different growth media selected for different bacterial isolates from the SML and the ULW samples, but MA yielded more diverse bacterial isolates. The cultivation of SML and ULW bacteria on MA resulted in the selection of 42 presumably marine bacteria, with the majority of isolates being Gram-negative. We also isolated and identified one potential pathogen (*Staphylococcus* sp. ZU-21) from the SML on MA (Table 1). Marine bacteria obtained from enriched SML samples were primarily identified as belonging to the *Bacillales* (10%), *Rhodobacterales* (14%), *Moraxellales* (24%), and *Alteromonadales* (52%) groups. Notable genera included *Thioclava*, *Psychrobacter*, and *Pseudoalteromona*s. Bacterial isolates obtained from the enriched ULW sample were identified as belonging to the *Micrococcales* (40%) and *Moraxellales* (60%) groups. Notable genera identified include *Glutamicibacter* and *Psychrobacter*. Most of the isolates (55) on MHA were Gram-positive, possible opportunistic pathogens (Supplementary Table S2), and were identified using MALDI-TOF. From SML samples, we retrieved bacterial isolates belonging to the *Bacillales* (22%), *Micrococcales* (61%), and *Hyphomicrobiales* (17%) groups. The representative genera were *Brevibacterium*, *Micrococcus*, and *Ochrobactrum*. From the ULW samples cultivated on MHA, we retrieved bacterial isolates belonging to the *Bacillales* (69%) and *Actinomycetes* (31%). The main representative genera were *Virgibacillus* and *Micrococcus* (see Supplementary Table S2).

**Table 1:** Bacterial isolates obtained from enriched SML and ULW samples on MA containing 100 ng/mL of ciprofloxacin and their phylogenetic affiliations.

Sample	Phylum	Bacterial class/order	Bacterial Isolate	Accession	Closest described relative	% Identity	Accession no
SML	*Bacillota*	*Bacilli/Bacillales*	*Metabacillus* sp. ZU-17	OR739683	*Metabacillus schmidteae* strain Marseille-P9898	99	NR_179484
			*Staphylococcus* sp. ZU-21	OR739685	*Staphylococcus saprophyticus* strain NBRC 102446	99	NR_114090
	*Pseudomonadota*	*Alphaproteobacteria/Rhodobacterales*	*Thioclava* sp. ZU-2	OR739665	*Thioclava dalianensis* strain DLFJ1-1 1	95	NR_118422
			*Thioclava* sp. ZU-10	OR739656	*Thioclava dalianensis* strain DLFJ1-1	95	NR_118422
			*Thioclava* sp. ZU-14	OR739659	*Thioclava nitratireducens* strain 25B10_4	95	NR_157648
	*Pseudomonadota*	*Gammaproteobacteria/Moraxellales*	*Psychrobacter* sp. ZU-1	OR739664	*Psychrobacter maritimus* strain Pi2-20	99	NR_027225
			*Psychrobacter* sp. ZU-15	OR739660	*Psychrobacter maritimus* strain Pi2-20	99	NR_027225
			*Psychrobacter* sp. ZU-8	OR739670	*Psychrobacter maritimus* strain Pi2-20	99	NR_027225
			*Psychrobacter* sp. ZU-16	OR739661	*Psychrobacter vallis* strain CMS 39	98	NR_042205
			*Psychrobacter* sp. ZU-3	OR739666	*Psychrobacter maritimus* strain Pi2-20	99	NR_027225
		*Gammaproteobacteria/Alteromonadales*	*Pseudoalteromonas* sp. ZU-20	OR739684	*Pseudoalteromonas neustonica* strain PAMC 28425	99	OQ626010
			*Pseudoalteromonas* sp. ZU-4	OR739667	*Pseudoalteromonas neustonica* strain PAMC 28425	99	OQ626010
			*Pseudoalteromonas* sp. ZU-9	OR739687	*Pseudoalteromonas neustonica* strain PAMC 28425	99	OQ626010
			*Pseudoalteromonas* sp. ZU-6	OR739686	*Pseudoalteromonas neustonica* strain PAMC 28425	99	OQ626010
			*Pseudoalteromonas* sp. ZU-5	OR739668	*Pseudoalteromonas ostreae* strain hOe-66 1	99	MT271987
			*Pseudoalteromonas* sp. ZU-18	OR739662	*Pseudoalteromonas ostreae* strain hOe-66	99	OQ472584
			*Pseudoalteromonas* sp. ZU-12	OR739658	*Pseudoalteromonas neustonica* strain PAMC 28425	99	OQ626010
			*Pseudoalteromonas* sp. ZU-19	OR739663	*Pseudoalteromonas prydzensis* strain MB8-11	98	NR_044803
			*Pseudoalteromonas* sp. ZU-7	OR739669	*Pseudoalteromonas prydzensis* strain MB8-11	99	NR_044803
			*Pseudoalteromonas* sp. ZU-11	OR739657	*Pseudoalteromonas ostreae* strain hOe-66	99	OQ472584
			*Pseudoalteromonas* sp. ZU-13	OR739682	*Pseudoalteromonas neustonica* strain PAMC 28425	99	OQ626010
ULW	*Actinomycetota*	*Actinomycetes/Micrococcales*	*Micrococcus* sp. FL-1	OR739676	*Micrococcus aloeverae* strain AE-6 16S	99	NR_134088
			*Glutamicibacter* sp. FL-2	OR739678	*Glutamicibacter bergerei* strain CIP 108036 (T)	99	MK424283
			*Glutamicibacter* sp. FL-5	OR739653	*Glutamicibacter bergerei* strain CIP 108036 (T)	99	MK424283
			*Glutamicibacter* sp. FL-12	OR739647	*Glutamicibacter bergerei* strain CIP 108036 (T)	100	MK424283
			*Glutamicibacter* sp. FL-19	OR739675	*Glutamicibacter ardleyensis* strain An25	99	NR_114901
			*Glutamicibacter* sp. FL-20	OR739651	*Glutamicibacter bergerei* strain CIP 108036 (T)	99	MK424283
			*Glutamicibacter* sp. FL-21	OR739677	*Glutamicibacter ardleyensis* strain CGMCC 1.3685	99	MT759863
			*Glutamicibacter* sp. FL-3	OR739652	*Glutamicibacter bergerei* strain Ca106	99	NR_025612
	*Pseudomonadota*	*Gammaproteobacteria/Moraxellales*	*Acinetobacter* sp. FL-11	OR739646	*Acinetobacter shaoyimingii* strain 323–1	97	MT138534
			*Psychrobacter* sp. FL-4	OR739679	*Psychrobacter maritimus* strain Pi2-20	98	NR_027225
			*Psychrobacter* sp. FL-6	OR739654	*Psychrobacter maritimus* strain Pi2-20	99	NR_027225
			*Psychrobacter* sp. FL-7	OR739655	*Psychrobacter maritimus* strain Pi2-20	99	NR_027225
			*Psychrobacter* sp. FL-8	OR739680	*Psychrobacter aquimaris* strain SW-210	99	NR_043140
			*Psychrobacter* sp. FL-9	OR739681	*Psychrobacter aquimaris* strain SW-21	99	NR_043140
			*Psychrobacter* sp. FL-10	OR739671	*Psychrobacter maritimus* strain Pi2-20	99	NR_027225
			*Psychrobacter* sp. FL-13	OR739672	*Psychrobacter namhaensis* strain SW-242	99	NR_043141
			*Psychrobacter* sp. FL-14	OR739673	*Psychrobacter pulmonis* strain CCUG 46240	98	NR_118026
			*Psychrobacter* sp. FL-15	OR739648	*Psychrobacter maritimus* strain Pi2-20	99	NR_027225
			*Psychrobacter* sp. FL-16	OR739674	*Psychrobacter maritimus* strain Pi2-20	99	NR_027225
			*Psychrobacter* sp. FL-17	OR739649	*Psychrobacter aquimaris* strain SW-210	98	NR_043140
			*Psychrobacter* sp. FL-18	OR739650	*Psychrobacter maritimus* strain Pi2-20	99	NR_027225

### 3.4 Bacterial communities across concentrations

**Figure 4:**
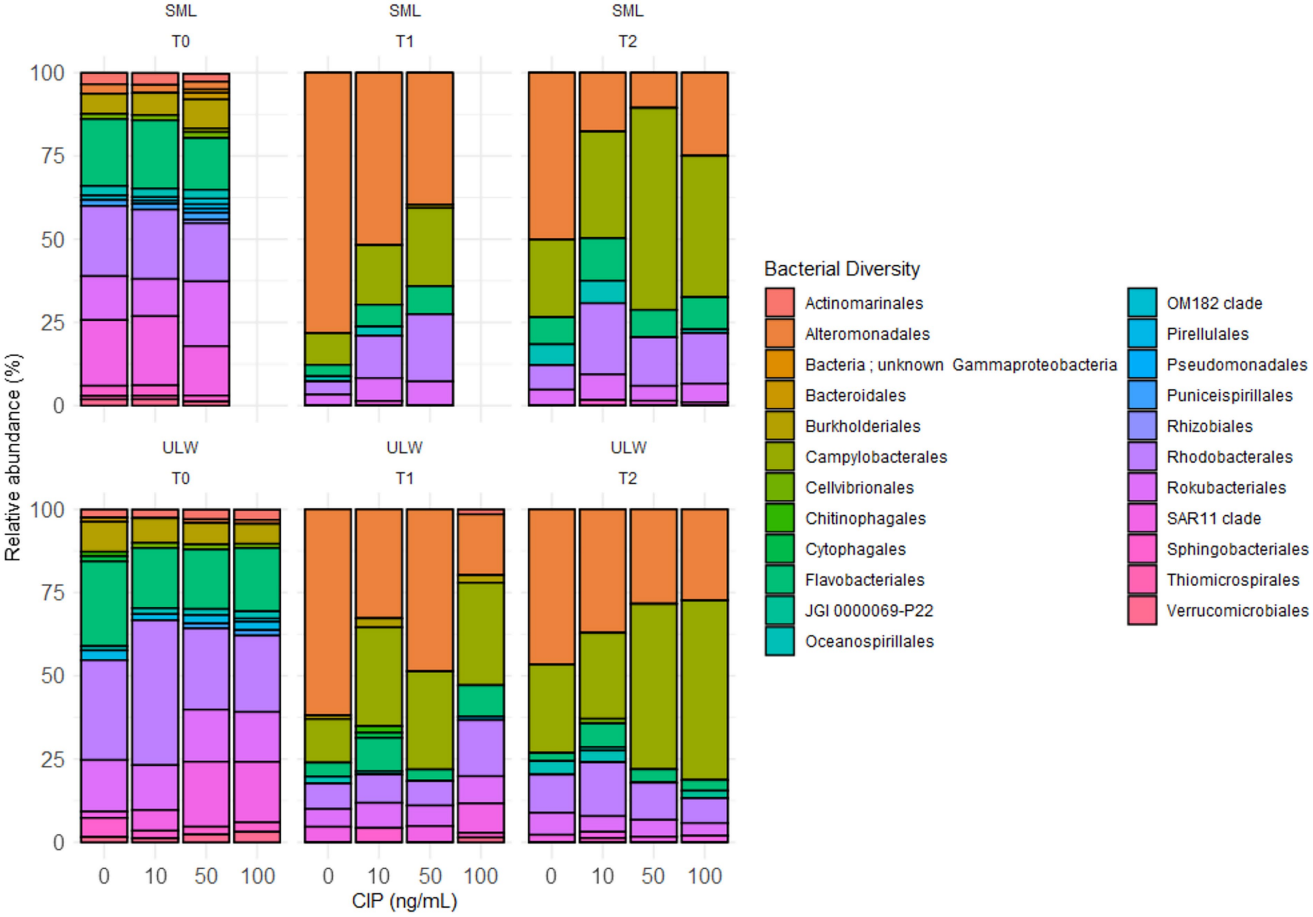
Bacterial community composition in the SML and the ULW across ciprofloxacin concentrations at different time points.

From the amplicon sequencing, we obtained a total of 2.3 × 10^6^ non-chimeric 16S rRNA genes, resulting in an average of 0.1 × 10^6^ sequence reads per sample. No significant differences were observed between the SML and ULW at any time points. The SML samples treated with 100 ng/mL and obtained at T0 and T1 were lost during the DNA extraction process. However, a higher number of 16S rRNA amplicon genes were recovered from the SML at T0, while more were retrieved from the ULW at T1 and T2 (Supplementary Figure S2a). There was also no significant difference between the 16S rRNA amplicon genes in the SML and ULW samples across concentrations (Supplementary Figure S2b). In total, we identified 9,578 ASVs across all concentrations and time points. At T0, ~ 17% of ASVs were shared among all ciprofloxacin concentrations (Supplementary Figure S3). At T1 and T2, the samples with of 0 ng/mL and 10 ng/mL ciprofloxacin had higher proportions of ASVs compared to the samples with higher concentrations. Approximately 18% of ASVs were shared across all concentrations at T0 in the ULW. The ULW samples with concentrations of 10 ng/mL, 50 ng/mL, and 100 ng/mL had higher proportions of ASVs at both T1 and T2 than the 0 ng/mL samples. The ASVs were distributed across 66 phyla, 380 orders, and 161 bacterial classes (see Supplementary Table S2), including candidate divisions. We obtained only bacterial communities with a relative abundance of at least 1%. Although ciprofloxacin was reported to have high activity against Gram-negative bacteria (Plumb, 2008), most bacterial communities sequenced in the SML and ULW were Gram-negative. The bacterial communities in both SML and ULW environmental samples at T0 were similar and composed of the bacterial orders *Flavobacteriales, Rhodobacterales*, and *Rhizobacterales*, as well as the SAR11 clade, with an average relative abundance of each exceeding 14%. However, these bacterial groups decreased at T1 and T2, i.e., their relative abundance was reduced at all concentrations, except for the *Rhodobacterales*, which had a higher relative abundance in SML samples with 10 ng/mL (21%), 50 ng/mL (15%), and 100 ng/mL (15%) ciprofloxacin compared to samples with 0 ng/mL (7%) at T2 (Figure 4).

The relative abundance of *Campylobacterales* and *Alteromonadales* strongly increased but also varied over time and with ciprofloxacin concentration. *Campylobacterales* increased with time and concentration in both the SML and ULW samples from ≤1% in the SML at T0 to 60% and 42% in samples with 50 ng/mL and 100 ng/mL at T2, respectively. In samples with 0 and 10 ng/mL ciprofloxacin, the relative abundance of *Campylobacterales* was 23 and 32%, respectively. In the ULW, a similar trend was observed, with the relative abundance of *Campylobacterales* shifting from 0% at T0 across all concentrations to about 30% in samples treated with 10, 50, and 100 ng/mL of ciprofloxacin at T1. By T2, the relative abundance of *Campylobacterales* in ULW samples with 50 ng/mL and 100 ng/mL ciprofloxacin was ≥ 50% and thus was much higher than in the other samples. At T0, the relative abundance of *Alteromonadales* was >3 and 2% in the SML and the ULW, respectively. By T1, the relative abundance of *Alteromonadales* in SML samples with 0 ng/mL increased to 78%, compared to their relative abundance in 10 ng/mL (52%) and 50 ng/mL (39%). Notably, at T2, the 100 ng/mL concentration showed a higher relative abundance of *Alteromonadales* compared to both the 10 and 50 ng/mL concentrations but decreased compared to T1. In the ULW, the relative abundance of *Alteromonadales* also increased to 62% in the 0 ng/mL, compared to 10 ng/mL (32%), 50 ng/mL (48%), and 100 ng/mL (18%) at T1. At T2, the relative abundance of *Alteromonadales* at 100 ng/mL was 27%, significantly lower than that at 0 ng/mL, which was 47%.

**Figure 5:**
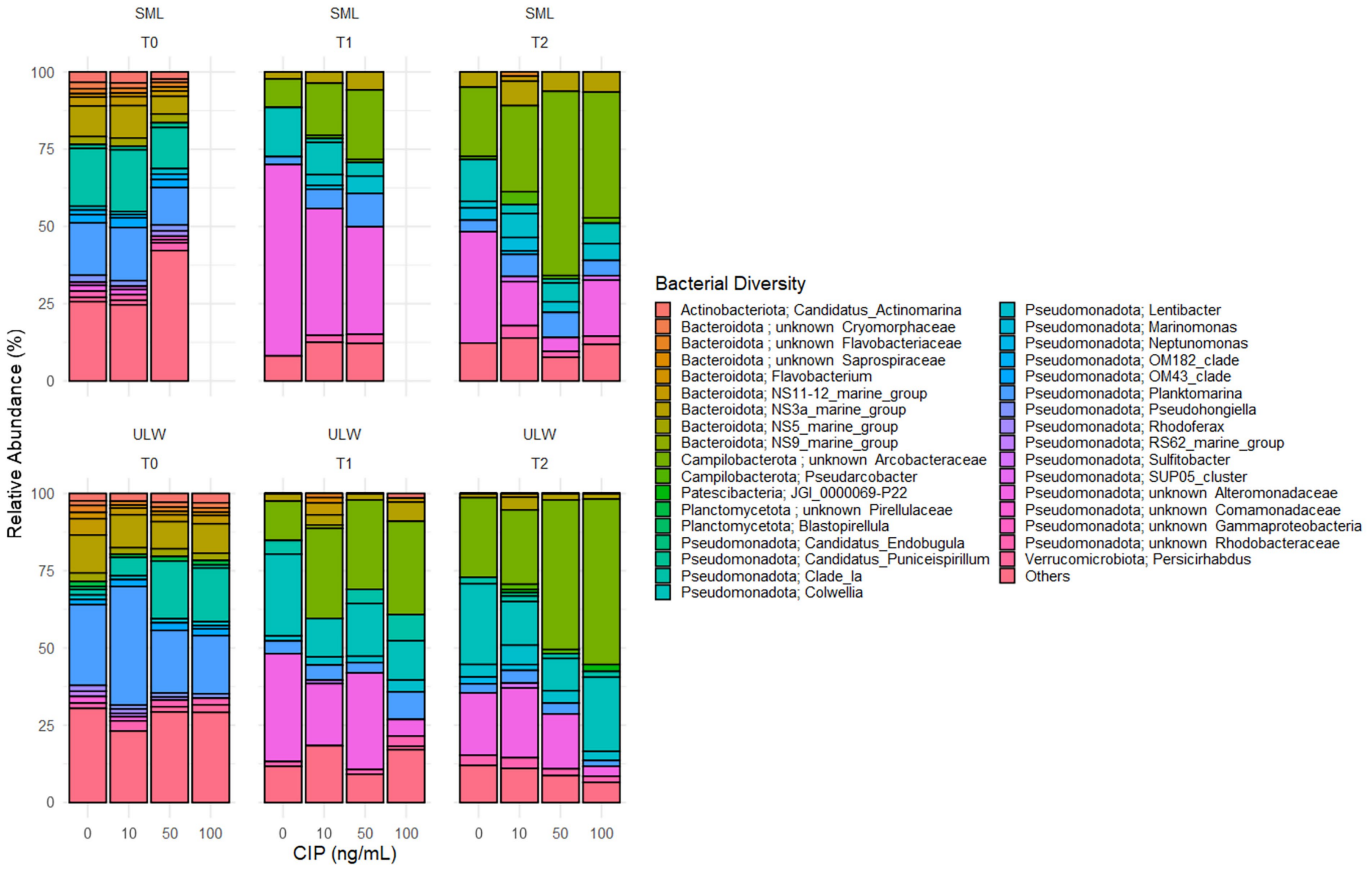
Composition of the bacterial community (genera/families) over time (T0, T1, and T2) in environmental SML and ULW samples treated with different ciprofloxacin concentrations. Bacterial diversity with a relative abundance of <0.5% are categorized as “others”.

A closer examination of the lower taxonomic ranks, specifically the genera (and sometimes families) of the bacterial communities, revealed that most genera exhibited a relative abundance of <0.5%, which were collectively classified as “others.” These rare communities account for more than 25% of the bacterial communities in both the SML and ULW at T0 (Figure 5). Among these are *Psychrobacter*, *Pseudoalteromona*s, *Acinetobacter,* and *Brevibacterium,* which were isolated in the presence of 100 ng/mL of ciprofloxacin. The relative abundances of these isolates ranged from 0.01 to 0.1% in the overall amplicon dataset. Bacterial genera/families with a relative abundance >1% at T0 are primarily marine species, including *Candidatus* Actinomarina*, Candidatus* Puniceispirillum*, Sulfitobacter*, *Pseudohongiella*, *Lentibacter,* and unknown *Rhodobacteraceae,* as well as marine groups NS11-12, NS3a, and NS5, with relative abundances ranging from 1 to 5% in both SML and ULW. By T1 and T2, the abundances of some of these groups had declined to 0%, regardless of the ciprofloxacin concentration. Some, such as *Lentibacter,* unknown *Rhodobacteraceae,* and NS3a (all *Rhodobacterales*), persisted until T1 and T2, irrespective of the ciprofloxacin concentration.

*Planktomarina* was also detected in both SML and ULW samples, showing abundances exceeding 12% in SML samples and more than 20% in ULW samples at T0. *Planktomarina* persisted until T1 and T2. At T2, their relative abundance decreased, particularly in the SML and ULW samples at 0 ng/mL. Notably, unknown *Arcobacteraceae* (*Campylobacterales*), initially not present at T0, became dominant in the SML and ULW samples, accounting for up to 60 and 40% in the SML samples containing 50 and 100 ng/mL of ciprofloxacin, respectively, as well as 48 and 53% in the ULW samples at the same concentrations. An increase in the relative abundance of unknown *Alteromonadaceae* was also observed, rising from 0% at T0, with the highest abundance found at T1. In addition, *Colwellia* (*Colwelliaceae, Alteromonadales*), initially present at 0% at T0, increased to 17 and 12% in the ULW samples containing 50 and 100 ng/mL, respectively, at T1. At T2, the relative abundance of *Colwellia* had increased to 24%.

### 3.5 Effects of ciprofloxacin on the bacterial community composition

**Figure 6:**
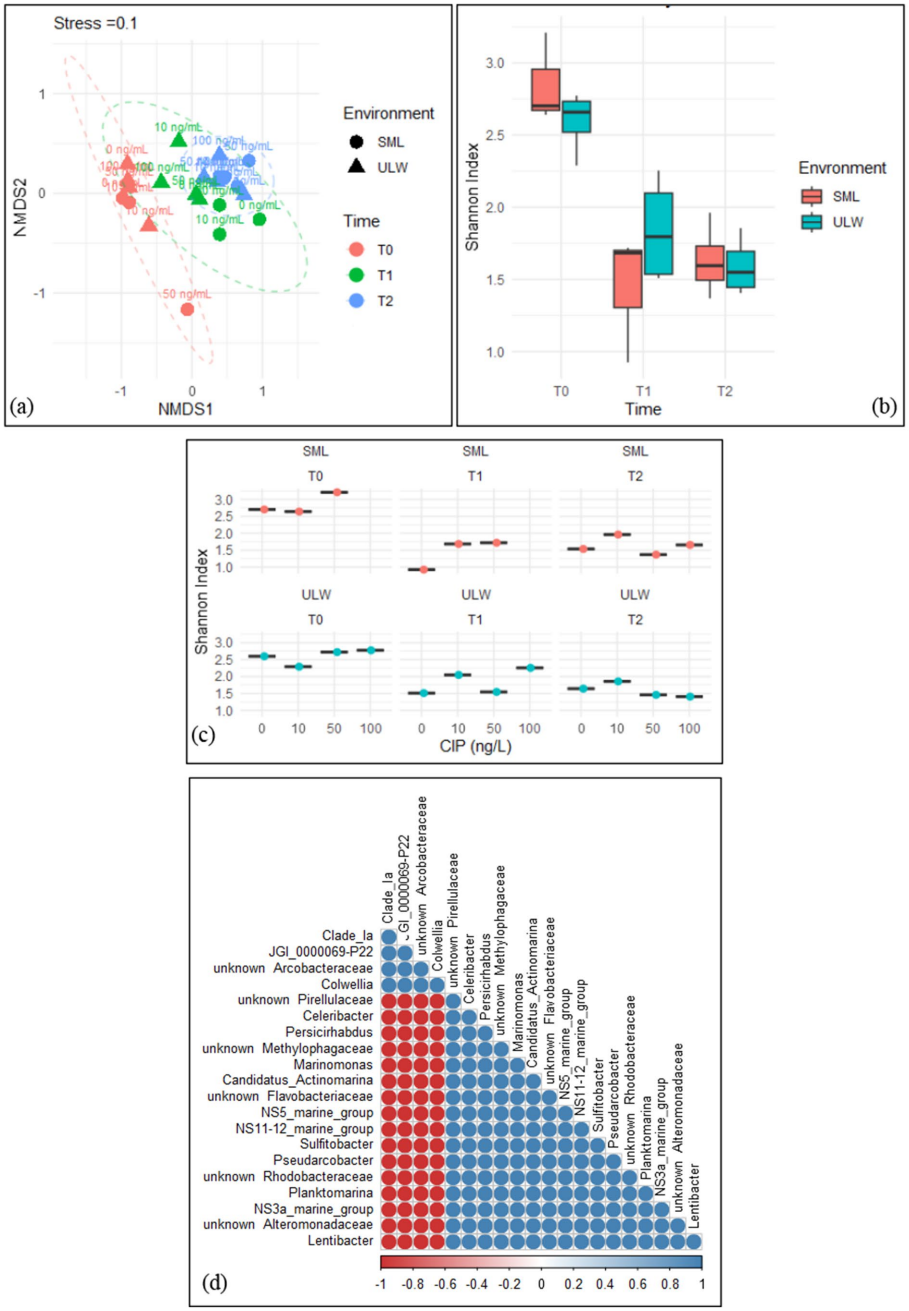
**(a)** Non-metric multidimensional scaling (NMDS) of the bacterial community composition in the SML and ULW samples across all ciprofloxacin concentrations and different time points. **(b)** Shannon index of the bacterial diversity within the SML and ULW samples at different time points. **(c)** Shannon index of the bacterial diversity of SML and ULW samples across the ciprofloxacin concentrations at different time points. **(d)** Correlation analysis of the 20 most abundant bacteria in the SML and ULW samples treated with 100 ng/mL ciprofloxacin and retrieved at T2. Red cycles indicate a strong negative correlation, while blue cycles indicate a strong positive correlation.

The bacterial communities within the SML and ULW were similar at T0. However, a significant shift in the community composition was observed at T1 and T2, irrespective of the ciprofloxacin concentrations (Figure 6a). We used PERMANOVA to statistically analyze the response of the bacterial communities in the SML and ULW to the different ciprofloxacin concentrations. The analysis revealed that time had a significant impact on the communities in the SML and ULW samples (*r*^2^ = 55%, *p* = 0.001). Notably, ciprofloxacin concentrations had no significant effect on the bacterial communities in either environment (*r*^2^ = 10%, *p* > 0.005) as the community composition shifted regardless of the concentrations. Nonetheless, the combination of both time and ciprofloxacin significantly influences bacterial community composition (*r*^2^ = 67%, *p* = 0.001), leading to the development of some bacterial taxa in response to ciprofloxacin.

Shannon’s diversity index indicated greater community diversity in the SML and ULW at T0, with more diversity within the SML compared to the ULW at T0 (Figure 6b), however, this difference was not statistically significant. Bacterial diversity decreased in both environments at T1 and T2. In addition, the bacterial communities within the SML and ULW samples treated with 10 ng/mL consistently exhibited slightly more diverse communities at T1 and T2 compared to samples without ciprofloxacin (Figure 6c). At T1, a more diverse bacterial community was observed in the ULW sample treated with 100 ng/mL than in those treated with lower concentrations. Pairwise correlation analysis categorized the 20 most abundant bacterial genera in the SML and ULW samples treated with 100 ng/mL at T2. The analysis showed only two distinct clusters (Figure 6d). The first cluster comprises the Clade_ Ia, Candidatus Bacterium JGI_0000069-P22, an unknown *Arcobacteraceae*, and *Colwellia*. All of these show strong positive correlations with each other, as indicated by the color gradient, i.e. The second cluster comprises marine bacteria, including *Lentibacter*, *Planktomarina, Sulfitobacter*, and an unknown *Rhodobacteraceae*, as well as other marine clades such as NS11-12, NS3a, and NS5. These bacteria also exhibit strong positive correlations with one another but strong negative correlations with Clade_ Ia, JGI_0000069-P22, an unknown *Arcobacteraceae,* and *Colwellia*.

### 3.6 Antibiotics in the SML/ULW

Two samples from the SML (SML_1 and SML_2) and two from ULW (ULW_1 and ULW_2) were analyzed for the presence of ciprofloxacin and 25 other antibiotics. Approximately twice the number of antibiotics detected in the ULW were found in the SML. Statistically, there was no significant difference in the concentration of antibiotics measured in the SML and ULW (*p* > 0.005). Still, novobiocin had the highest concentration in the SML (16.92 ng/L) and ULW (3.99 ng/L). We detected a maximum of 11 antibiotics in the samples obtained from the SML, while only six were detected in the samples collected from the corresponding ULW. Of the antibiotics analyzed, ciprofloxacin was not detected. However, erythromycin, lincomycin, trimethoprim, sulfamethoxazole, roxithromycin, chloramphenicol, clarithromycin, ofloxacin, clindamycin, tylosin, and novobiocin were found in the environmental samples. Chloramphenicol, roxithromycin, sulfamethoxazole, trimethoprim, and erythromycin were found only in the SML samples and not the ULW samples (Table 2).

**Table 2:** Target antibiotics in the SML and ULW.

Antibiotic class	Antibiotic type	SML_1	SML_2	ULW_1	ULW_2
Lincosamides	Lincomycin (+)	0.08	0.11	0.06	0.15
	Clindamycin (+)	0.68	1.12	1.03	1.08
	Clarithromycin (+)	1.07	0.21	0.22	0.20
Macrolides	Spiramycin (+)	<LoD	<LoD	<LoD	<LoD
	Tylosin (+)	5.08	1.52	3.48	3.07
	Erythromycin (+)	0.14	0.10	<LoD	<LoD
	Azithromycin (+)	<LoD	<LoD	<LoD	<LoD
Quinolones	Ofloxacin (+)	0.39	1.35	1.79	<LoQ
	Enrofloxacin (+)	<LoD	<LoD	<LoD	<LoD
	Roxithromycin (+)	0.76	0.18	<LoD	<LoD
	Ciprofloxacin (+)	<LoD	<LoD	<LoD	<LoD
	Sparfloxacin (+)	<LoD	<LoD	<LoD	<LoD
Phenicol	Chloramphenicol (−)	1.00	0.58	<LoD	<LoD
Sulfonamides	Sulfadiazine (+)	<LoD	<LoD	<LoD	<LoD
	Sulfadimethoxine (+)	<LoD	<LoD	<LoD	<LoD
	Sulfamethazine (+)	<LoD	<LoD	<LoD	<LoD
	Sulfamethizole (+)	<LoD	<LoD	<LoD	<LoD
	Sulfamethoxazole (+)	0.26	0.31	<LoD	<LoD
	Sulfaguanidine (+)	<LoD	<LoD	<LoD	<LoD
	Sulfathiazole (+)	<LoD	<LoD	<LoD	<LoD
Aminocoumarin	Novobiocin (+)	5.64	16.92	1.19	3.99
Tetracyclines	Doxycycline (+)	<LoD	<LoD	<LoD	<LoD
	Chlortetracycline (+)	<LoD	<LoD	<LoD	<LoD
	Oxytetracycline (+)	<LoD	<LoD	<LoD	<LoD
Diaminopyrimidines	Trimethoprim (+)	0.76	0.18	<LoD	<LoD
Rifamycin	Rifampicin (+)	<LoD	<LoD	<LoD	<LoD

All antibiotics, except chloramphenicol, were analyzed under positive electrospray ionization (ESI+) modes. LoD, limit of detection; LoQ, limit of quantification.

### 3.7 Resistance profiles of enriched bacteria to antibiotics

**Figure 7:**
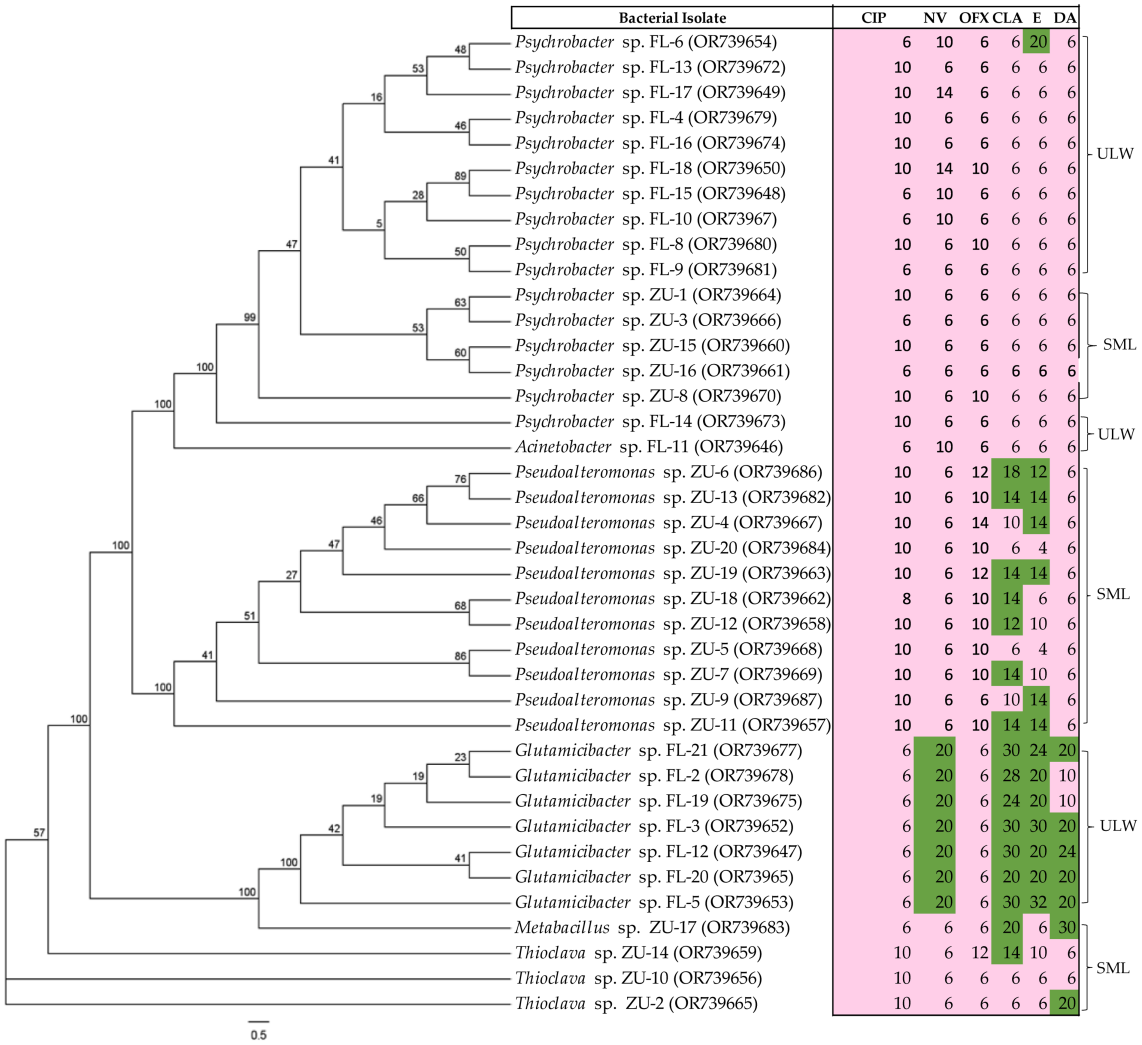
Antibiotic susceptibility patterns of the marine bacterial isolates obtained from the SML and ULW. Isolates were tested on MA. The inhibition zones are given in mm for antibiotic susceptibility testing and were interpreted using modified EUCAST breakpoints, with susceptible shown in green and resistant in pink. For ciprofloxacin (CIP), inhibition zones <22 mm = resistant; novobiocin (NV), <16 mm = resistant; ofloxacin (OFX), <22 mm = resistant; clarithromycin (CLR) and erythromycin (E), <12 mm = resistant; clindamycin (DA), <17 mm = resistant. The values on each node of the tree indicate the bootstrap percentage supporting the inferred relationships among bacterial isolates.

A total of 39 bacterial strains from enriched SML and ULW samples, cultivated on MA, were tested against ciprofloxacin, novobiocin, ofloxacin, clarithromycin, erythromycin, and clindamycin. We intentionally excluded potential or opportunistic pathogens and primarily non-marine organisms during the antibiotic susceptibility tests. As expected, the majority of the cultivated bacteria showed high resistance to most of the antibiotics tested (Figure 7). Similar bacterial genera also exhibited comparable resistance patterns. For example, 16 *Psychrobacter* spp. isolated from SML and ULW-enriched samples demonstrated resistance to all tested antibiotics, particularly ofloxacin, clarithromycin, erythromycin, and clindamycin. Additionally, 11 *Pseudoalteromonas* spp. isolated from the SML-enriched samples also displayed widespread resistance to ciprofloxacin and ofloxacin; however, 7 and 6 *Pseudoalteromonas* spp. were found to be susceptible to clarithromycin and erythromycin, respectively. Lastly, 7 *Glutamicibacter* spp., which are Gram-positive bacteria mainly cultivated from ULW samples, exhibited greater susceptibility to novobiocin, clarithromycin, erythromycin, and clindamycin, while only one *Thioclava* spp. was susceptible to clindamycin out of the three *Thioclava* spp. isolated from enriched SML samples.

## 4 Discussion

### 4.1 Bacterial cell counts and enrichment are significantly affected by high ciprofloxacin concentrations

Bacterial abundance was significantly enriched in SML samples obtained at T1, irrespective of ciprofloxacin concentrations, as the EF was ≥1. The environment is heterotrophic, allowing for the high proliferation of bacterial communities (Wurl et al., 2011). The availability of dissolved organic matter and the accumulation of biofilm matrix in the SML serve as a significant hotspot for bacterial proliferation. Our SML samples appear more turbid than our ULW samples, which suggests the possibility of organic matter accumulation. The competition for available nutrients probably resulted in a sharp decrease in bacterial abundance at T2 in SML samples (see Figure 2a). This sharp decrease is unlikely to be attributed to the effect of ciprofloxacin, as bacterial abundance was still higher in the SML samples treated with 100 ng/mL. Most of the decrease occurred in SML samples containing no antibiotic (0 ng/mL) and lower antibiotic concentrations (10 and 50 ng/mL), supporting the idea that high concentrations led to the growth of bacteria from the SML and ULW, which were already resistant to ciprofloxacin.

Ciprofloxacin concentrations of 100 ng/mL demonstrated a significant impact on bacterial cell numbers in our SML/ULW samples compared to low concentrations (0 and 10 ng/mL). This effect is further substantiated by our growth experiments, which showed lower growth at these concentrations. Ciprofloxacin concentrations ranging from 10 to 100 ng/mL have been shown to impact bacterial cells in various aquatic ecosystems (Flores-Vargas et al., 2023; Marti et al., 2016). Nonetheless, we observed that bacterial cell counts and growth increased linearly over time at these concentrations, indicating that ciprofloxacin exerted a selective pressure on SML and ULW bacteria. This selective pressure resulted in the emergence of resistant organisms within 7 days of incubation, probably due to the acquisition of ciprofloxacin resistance genes. This is because ciprofloxacin concentrations of 10–100 ng/mL were shown to significantly increase the copy numbers of quinolone resistance determinants, such as the *qnrS* gene, in a pristine aquatic environment within 24 h (Marti et al., 2016).

These resistance determinants confer resistance to ciprofloxacin and other quinolones. Our sampling site is not pristine, being characterized by various anthropogenic activities, including wastewater effluent discharge (Adenaya et al., 2024b). Nonetheless, other quinolone resistance determinants such as *qsrA* and *qsrB* have been identified in marine bacteria obtained in a coastal environment close to aquaculture farms, where the use of antibiotics is prevalent (Allou et al., 2009). This suggests that ciprofloxacin has the potential to contribute to antibiotic resistance in our environmental samples. This is important, as bacterial growth in a 100 ng/mL concentration of ciprofloxacin continues to rise linearly for 7 days (see Figure 3), thereby supporting the emergence and dominance of resistant bacteria. Bacterial cells can undergo rapid adaptive responses to ciprofloxacin by mutating at a mutation rate of 2.6 × 10^6^ per cell division under ciprofloxacin pressure, in a phenomenon known as growth-dependent mutation (Riesenfeld et al., 1997). Bacterial phenotypic adaptation can occur as soon as one hour after exposure to ciprofloxacin (Soares et al., 2019).

Based on our results, it seems obvious that treatment with 100 ng/mL ciprofloxacin eliminated susceptible marine bacteria and left resistant ones behind. The continuous exposure of the bacteria during cultivation on MA to 100 ng/mL of ciprofloxacin led primarily to the selection of strains affiliated with known groups of marine bacteria. Most of these perform essential functions in coastal environments (e.g., *Psychrobacter, Pseudoalteromonas*, and *Thioclava* spp.) (Romanenko et al., 2004). *Acinetobacter, Glutamicibacter*, *Metabacillus*, and various species of *Staphylococcus* have been identified across diverse environments, including aquatic systems, terrestrial soils, food sources, and animal hosts. These findings are documented within the BacDive database, which provides comprehensive information regarding the diversity and origin of bacteria (Reimer et al., 2022). Most of them can withstand high salt concentrations; hence, they were isolated on MA. In our comprehensive amplicon analysis, we observed a notable presence of *Psychrobacter* and *Pseudoalteromonas,* indicating their prevalence in the environment, albeit in low abundance. *Psychrobacter* and *Pseudoalteromonas* were also the most abundant bacterial isolates in our cultivation, with 100 ng/mL on MA (Table 1). This suggests that under an ideal physiological condition of marine environments, such as availability of various salts, vitamins, amino acids, and other essential components, as well as favorable temperatures ranging from 15°C to 20°C (typical during summer), they are likely to proliferate in areas prone to contaminants such as antibiotics.

The continuous cultivation of bacteria on MHA with 100 ng/mL of ciprofloxacin also eliminated susceptible bacteria but led to the selection of non-marine bacteria. MHA does not allow the growth of typical marine bacteria but supports the growth of pathogens (Silva et al., 2022). Accordingly, some bacteria cultivated on MHA with 100 ng/mL ciprofloxacin appear to be opportunistic pathogens (e.g., *Bacillus cereus, Micrococcus luteus, Brevibacterium*), capable of causing infections (Griffiths and Schraft, 2017; Shweta et al., 2021). Some organisms closely related to our new strains are ubiquitous in food, such as milk and cheese (Anast et al., 2019). The isolation of these bacteria and possible pathogens from our SML and ULW enriched samples might be explained by the inlets of wastewater surrounding our sampling site. The bacterial isolates were not represented in our amplicon dataset, supporting their non-marine origins and the possibility of their development under the right physiological conditions.

### 4.2 Marine bacterial community dynamics in response to ciprofloxacin exposure

The dominant marine taxa across all ciprofloxacin concentrations and time points include *Alteromonadales*, *Flavobacteriales*, *Oceanospirillales*, and *Rhodobacterales*. The relative abundance of these bacterial communities increased over time, regardless of ciprofloxacin concentration, suggesting their resistance and degradation capabilities. The 100 ng/mL concentration is higher than the minimum inhibitory ciprofloxacin concentration against some clinical bacteria, such as *Enterobacterale*s (60 ng/mL) and *Haemophilus* (60 ng/mL) (EUCAST, 2024). Hence, 100 ng/mL ciprofloxacin can inhibit or kill specific clinical pathogens, while some bacteria in the SML and ULW can survive or persist at this concentration. This supports the idea that resistance patterns might be at play, where marine bacteria can withstand high levels of antibiotics typically used in clinical settings. This finding also supports our previous study (Adenaya et al., 2024a), which demonstrated that several marine bacteria isolated from the SML of the same sampling area exhibit multiple forms of antibiotic resistance.

Indeed, two bacterial strains belonging to the order *Rhodobacterales* harbored multiple antibiotic resistance genes, indicating a significant capacity for resistance to a variety of antibiotics, as evidenced by physiological assessments (Adenaya et al., 2024a). Abundance over time also suggests that these taxa may possess enzymatic pathways or metabolic processes effective in breaking down ciprofloxacin, thus enabling their survival and growth in environments contaminated with ciprofloxacin. Hence, the persistence and resilience of *Alteromonadales, Flavobacteriales*, *Oceanospirillales*, and *Rhodobacterales* across all ciprofloxacin concentrations highlight their ecological importance in the degradation of organic compounds (Bunse and Pinhassi, 2017) and potential utility in bioremediation strategies aimed at mitigating the impacts of pharmaceutical pollution in coastal ecosystems.

The communities classified under *Campylobacterales*, particularly unknown *Arcobacteraceae,* exhibited distinct trends. At T0, the relative abundance of these microbial taxa ranged between 1 to 3% in both SML and UWL. However, there was an increase in their prevalence over time, which corresponded with higher concentrations of ciprofloxacin. This finding is not unexpected, as *Arcobacteraceae* are known to possess putative genes that degrade various organic compounds (Li et al., 2024). Members of the *Arcobacteraceae* primarily originate from terrestrial habitats, including human and animal intestines, as well as wastewater treatment plants (Li et al., 2024; Venâncio et al., 2022). Therefore, their low relative abundance at T0 suggests that they were already present in the environment. However, their prevalence in the presence of high ciprofloxacin raises concerns, indicating that it could enhance their abundance and spread if present in the environment. This is important due to the pathogenic nature of certain members of the *Arcobacteraceae*, which exhibit a widespread distribution at the animal-human-environment interface (Buzzanca et al., 2023). The enhanced prevalence of *Arcobacteraceae* in response to ciprofloxacin exposure thus suggests a potential risk of increased pathogenicity in coastal ecosystems. Furthermore, given that some species within this family have been associated with foodborne illnesses and infections (Venâncio et al., 2022), their abundance in environmental samples over time and concentration shows that they could pose significant public health challenges.

### 4.3 Other factors could contribute to the bacterial community’s response to ciprofloxacin exposure

While high concentrations of ciprofloxacin, specifically 50 and 100 ng/mL, affected bacterial cell counts and enrichment, significant changes in bacterial abundance and diversity were observed at various time points, regardless of the ciprofloxacin concentration. This suggests that temporal factors, in addition to ciprofloxacin, played a crucial role in shaping community dynamics. Furthermore, it indicates that other variables, such as nutrient depletion, nutrient release from dead or lysed cells, hormesis, competition for available resources, spatial structuring, and interactions with other microbial species, may be driving the observed changes in bacterial abundance and diversity in the SML and ULW samples over time. The observed linear increase in bacterial cell counts and growth across all concentrations of ciprofloxacin indicates that nutrient depletion is unlikely to be a contributing factor. Bacteria can utilize nutrients released from dead biomass (Hanajima et al., 2019). Additionally, the bacterial communities may be experiencing hormesis, which could enhance their resilience, particularly at low concentrations of ciprofloxacin (Iavicoli et al., 2021). In any case, the observed shifts in bacterial communities at time points T1 and T2 (Figure 6a) indicate a reorganization that favors the dominance of specific taxa while concurrently excluding others across all ciprofloxacin concentrations, including samples without ciprofloxacin. This phenomenon suggests that competition may be a significant factor in the dynamics we observed from T0 to T1 and T2.

Competition operates in a ‘kill-or-be-killed’ manner, resulting in shifts in bacterial communities over time (Hibbing et al., 2010). Our samples were continuously mixed without the addition of nutrients for a period of 7 days. During this period, bacterial groups possessing similar nutrient requirements may have engaged in competitive interactions for the acquisition of the limited nutrients taken up by faster-growing populations (Hibbing et al., 2010). Typically, bacteria with high growth rates exhibit increased substrate affinity, thereby enhancing their competitive fitness (Carrero-Colón et al., 2006). This, in turn, influences bacterial responses to environmental factors over time (Carrero-Colón et al., 2006). Such competition likely involves interactive strategies that necessitate cooperation among bacterial populations of closely related taxa. It is therefore not surprising that the most dominant bacterial taxa we observed at T1 and T2 across all ciprofloxacin concentrations are typically marine taxa known for their competition prowess.

This is supported by correlation analysis, which reveals two clusters, with one cluster containing several taxa that are positively correlated in 100 ng/mL of ciprofloxacin at T2 (see Figure 6d). This cluster comprises several marine bacteria, including *Lentibacter*, *Planktomarina*, and *Sulfitobacter*, all of which belong to the order *Rhodobacterales*. These taxa are well-known for their competitive fitness and their ability to produce secondary metabolites, such as terpenes and bacteriocins, in marine environments (Huang et al., 2023). Typically, secondary metabolites are produced in response to antibiotic stress (Li et al., 2020). Thus, it is likely that ciprofloxacin triggered a stress response in these bacterial isolates, contributing to their persistence over time. This observation is further supported by the finding that the relative abundance of *Rhodobacteriales* was higher in samples containing ciprofloxacin compared to those without, particularly in the SML (see Figure 4). Additionally, this is corroborated by statistical analysis, which indicated that the combination of time and ciprofloxacin significantly influenced the dynamics of the bacterial community in the SML and ULW samples.

### 4.4 The SML had more antibiotics than the ULW

The presence of antibiotics in coastal environments is a significant global health concern. We detected 11 and 6 antibiotics in samples obtained from SML and ULW samples, respectively, even though the samples were taken from the same sampling point. Among all the antibiotics detected in the environment, chloramphenicol, roxithromycin, sulfamethoxazole, trimethoprim, and erythromycin were detected only in the SML and were absent in the ULW samples (see Table 2). Also, the concentrations of antibiotics in the SML were higher than those measured in the ULW. Surface-active substances pertinent to the SML may enable the environment to act like a membrane, accumulating contaminants introduced into water bodies (Chen et al., 2023). Additionally, pharmaceutical contaminants, such as antibiotics, may have a higher affinity for the SML than for the ULW environment (Adenaya et al., 2023a,b). The molecular structures of some antibiotics feature hydrophobic groups such as aromatic rings, alkyl chains, and other non-polar side chains.

These groups may enhance the adsorption of antibiotics on water surfaces (Kim et al., 2023). For example, chloramphenicol, a hydrophobic antibiotic containing a nitrobenzene ring (Kim et al., 2023), is likely to be detected primarily in the SML due to its complete hydrophobicity. Erythromycin and roxithromycin also have hydrophobic regions that enable the antibiotics to interact with lipid membranes and proteins (Zieliński et al., 2021). The SML has high concentrations of organic matter, including lipids, fatty acids, and proteins (Alpert et al., 2017). These can bind to hydrophobic antibiotics and facilitate their accumulation in the SML. Further studies are, however, needed to investigate the characteristics of antibiotics and the factors that promote their affinity to the SML compared to the corresponding ULW. We did not detect ciprofloxacin, but we did detect ofloxacin in the SML and ULW. The investigation into the impact of ciprofloxacin on SML and ULW bacterial dynamics commenced before the chemical analysis. However, the absence of ciprofloxacin in our samples does not negate its widespread presence in other coastal waters influenced by anthropogenic inputs (Biel-Maeso et al., 2018). Approximately 60% of ciprofloxacin is excreted, and concentrations in influents and effluents could be up to 2,831 and 564 ng/L, respectively (Biel-Maeso et al., 2018; Šíma et al., 2022). The absence of ciprofloxacin detection in our environmental samples can be attributed to several factors, including photochemical and biological degradation (Guo et al., 2013; Mohy-u-Din et al., 2024).

Ciprofloxacin may continue to undergo photochemical degradation due to strong exposure of the SML to ultraviolet (UV) radiation and temperature fluctuations. UV radiation can increase the concentration of natural oxidants, such as hydrogen peroxide (H₂O₂), which is generated through photochemical reactions in seawater, especially in the SML (Walczak and Swiontek Brzezinska, 2009). Indeed, Guo et al. (2013) have shown that UV/H₂O₂ yields 16 degradation products of ciprofloxacin, indicating a more extensive degradation process, particularly at pH 7.0. The samples for this study were collected in March (the beginning of spring) with ample sunlight and an ambient air temperature of approximately 10°C. The pH of our sampling site was 7.8 (see Section 2.1), which is slightly higher but still conducive to effective photodegradation (Torniainen et al., 1996), particularly in the SML and subsurface water (low-medium turbidity). Since the antibiotic in most cases exists in a zwitterionic form at pH levels above 6, lower concentrations are photolyzed more readily than higher concentrations, following first-order kinetics (Torniainen et al., 1996; Guo et al., 2013). Thus, on the one hand, the relatively rapid rates of photodegradation, especially under UV/H₂O₂ conditions, may account for the absence of ciprofloxacin in both the SML and the water 1 m below, i.e., the ULW, as it could have been degraded before accumulating. On the other hand, ciprofloxacin concentrations might decrease to levels below the detection threshold, defined by the LC–MS method as less than 0.001 ng/L (Adenaya et al., 2023b). Nonetheless, further research is needed to examine the concentrations of antibiotics in the SML and the ULW, particularly during the summer months, when sunlight is mostly abundant, compared to winter months, when sunlight availability is significantly reduced.

Biological degradation occurs through the proliferation of ciprofloxacin-resistant bacteria, resulting in a removal efficiency of approximately 90% (Mohy-u-Din et al., 2024). Thus, biological degradation could also play a significant role in the removal of antibiotics in the environment, as our findings indicate that SML and ULW are inhabited by bacterial species known for their biodegradation capabilities. For example, the marine bacterium *Lentibacter*, which dominated our amplicon dataset across all ciprofloxacin concentrations at different time points, exhibits the ability to degrade a diverse array of organic compounds, including complex hydrocarbons in marine environments (Vázquez Rosas Landa et al., 2023). *Psychrobacter* spp. that were found to be enriched in our study are present in anthropogenically influenced coastal environments due to their ability to degrade pollutants and utilize various organic compounds (Liu et al., 2023). The metabolic versatility of these bacteria positions them as a potential contributor to bioremediation processes and the ecological cycling of organic materials in marine environments. However, confirming biodegradation in our study will necessitate measuring the disappearance of ciprofloxacin at various time points and along gradients, which falls outside the scope of this research. Moreover, ofloxacin was detected. Ofloxacin belongs to the fluoroquinolone class of antibiotics and shares comparable resistance mechanisms and genetic determinants with ciprofloxacin (Amel et al., 2024). Consequently, the administration of ofloxacin may also contribute to the development of ciprofloxacin resistance, as evidenced by observations in clinical isolates (Amel et al., 2024; Serwacki et al., 2024).

### 4.5 Enriched bacteria showed resistance to antibiotics found in the environment

Based on the results of the chemical analysis, we investigated the susceptibility of our marine isolates from the SML and ULW to clinically important antibiotics, including clarithromycin, erythromycin, clindamycin, novobiocin, and ofloxacin, which are commonly found in the environment. This part of our study has several limitations, as there are currently no established methods, breakpoints, or media for testing the antibiotic susceptibility of marine bacteria. Therefore, MA was used for the antibiotic susceptibility tests, and breakpoints from clinically relevant bacteria (*Enterobacterales*, *Vibrio*, and *Bacillu*s spp.) were applied. In the case of novobiocin, we utilized an arbitrary breakpoint. Whether these breakpoints and the disk diffusion methodology are suitable for marine bacteria is unknown. Therefore, the resistance observed has to be interpreted with caution. Our findings indicate that marine bacteria exhibit widespread resistance to antibiotics under environmental conditions relevant to their natural habitat. This is not surprising because the bacterial isolates had been subjected to antibiotic stress, enabling them to develop resistance to the pressure imposed by antibiotics. In addition, the limited susceptibility of marine bacteria to various antibiotics, including ampicillin, erythromycin, gentamicin, tetracycline, sulfadimidine, kanamycin, and penicillin, has been reported (Nieto et al., 1993; Adenaya et al., 2024b). *Psychrobacter* spp. is the only genus isolated in both the SML and ULW despite continuous treatment with 100 ng/mL ciprofloxacin (Table 1). The isolates are also represented in the amplicon dataset across all concentrations at all time points. *Psychrobacter* spp. showed widespread resistance to all antibiotics, particularly to those found in the environment (Figure 7). Thus, the selective pressure based on the presence of antibiotics in environmental samples may increase the prevalence of antibiotic-resistant bacteria, raising concerns about the continuous discharge of antibiotics and the potential development and spread of antibiotic resistance in coastal environments.

## 5 Conclusion

To the best of our knowledge, our study is the first to directly assess the potential impact of antibiotics in the SML and ULW of a representative coastal environment influenced by anthropogenic activities. It, therefore, fills a knowledge gap and contributes to understanding the dynamics of marine bacterial communities and their varied responses to external factors, such as antibiotics in coastal ecosystems, over time. Coastal ecosystems act as a reservoir for anthropogenic contaminants, including antibiotics, which influence the resistance and adaptability of marine bacteria. Antibiotics are continuously infiltrating coastal environments, necessitating an investigation into their impact on marine bacterial dynamics over time. Our study showed that ciprofloxacin concentration and temporal dynamics can have a significant effect on bacterial community structures in coastal ecosystems affected by anthropogenic activities. In SML/ULW samples, bacterial cell counts and growth displayed a consistent linear increase over time, even at higher concentrations of ciprofloxacin. Notably, bacterial growth in samples treated with the highest concentration of 100 ng/mL continued to increase over 7 days without reaching a peak, indicating the enrichment of bacteria that were already resistant to the antibiotic. This observation is particularly important due to continuous interactions occurring between marine and pathogenic bacterial communities in marine environments (Gnanagobal and Santander, 2022). For instance, the *Arcobacteraceae* family, which exhibited a high relative abundance in the SML/ULW samples treated with high concentrations of ciprofloxacin at T1 and T2, comprises several potential pathogens (Buzzanca et al., 2023). Indeed, many marine bacterial communities were identified in these samples, with some exhibiting an increase over time in response to higher ciprofloxacin concentrations, similar to those of the *Arcobacteraceae*. This indicates the possibility of complex interactions, where antibiotic-resistant determinants may be exchanged through horizontal gene transfer. The potential for the exchange of antibiotic resistance determinants among diverse bacterial populations raises important implications for understanding microbial ecology and public health. While ciprofloxacin was not detected in this study, ofloxacin, which belongs to the same class of antibiotics and has a similar mode of action and resistance determinant to ciprofloxacin, was detected. Other relevant antibiotics, such as clindamycin, clarithromycin, and erythromycin, with widespread resistance, were also detected, indicating environmental exposure to anthropogenic pollutants. Our study thus highlights the need for intensified efforts to safeguard both coastal ecosystems and public health.

## Data Availability

The original contributions presented in the study are publicly available. This data can be found: https://doi.pangaea.de/10.1594/PANGAEA.974345 and the European Nucleotide Archive under the accession number PRJEB91248 (https://www.ebi.ac.uk/ena/browser/view/PRJEB91248).

## Footnotes

^1^ https://bioinfogp.cnb.csic.es/tools/venny
