# Supplementary material for: Effects of ciprofloxacin on bacterial abundance and enrichments in samples taken from the sea surface microlayer and underlying waters in the southern North Sea
Source: Front Microbiol. 2025 Aug 27;16:1624041. doi: 10.3389/fmicb.2025.1624041 (PMC12421915; doi:10.3389/fmicb.2025.1624041)
Supplement: Supplementary file 1 [file Supplementary_file_1.docx]

Supplementary Material


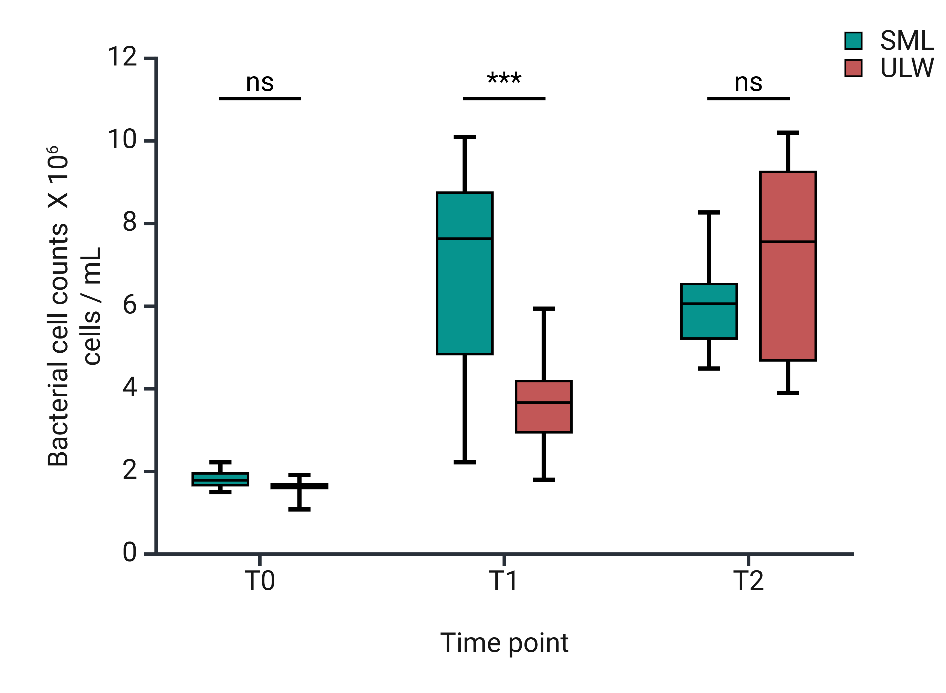


Figure S1. The difference in bacterial cell counts across various time points. The total cell counts of all bacterial species at each concentration of ciprofloxacin. Each box plot displays the median cell count at each time point, allowing for a comparison of the differences observed between the SML and ULW at different time points under different ciprofloxacin concentrations. Asterisks (P < 0.001), “ns" (significant difference). T0 samples were taken on the sampling day, T1 samples after three days, and T2 samples after seven days of incubation.


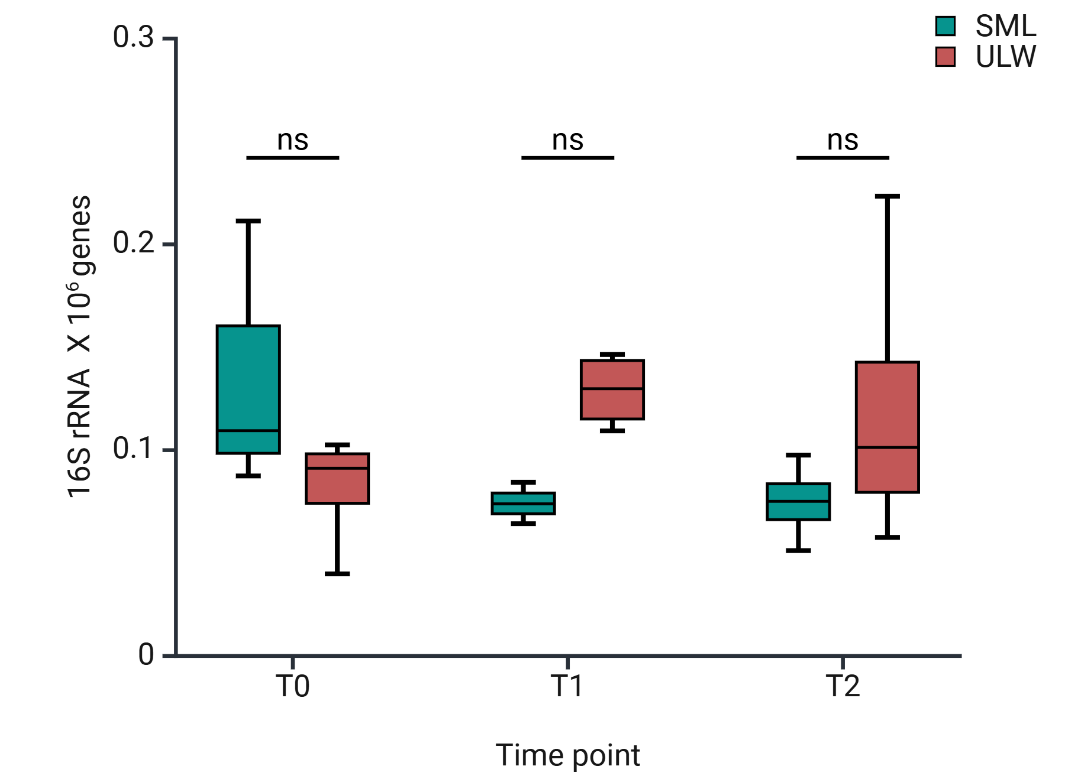


(a)


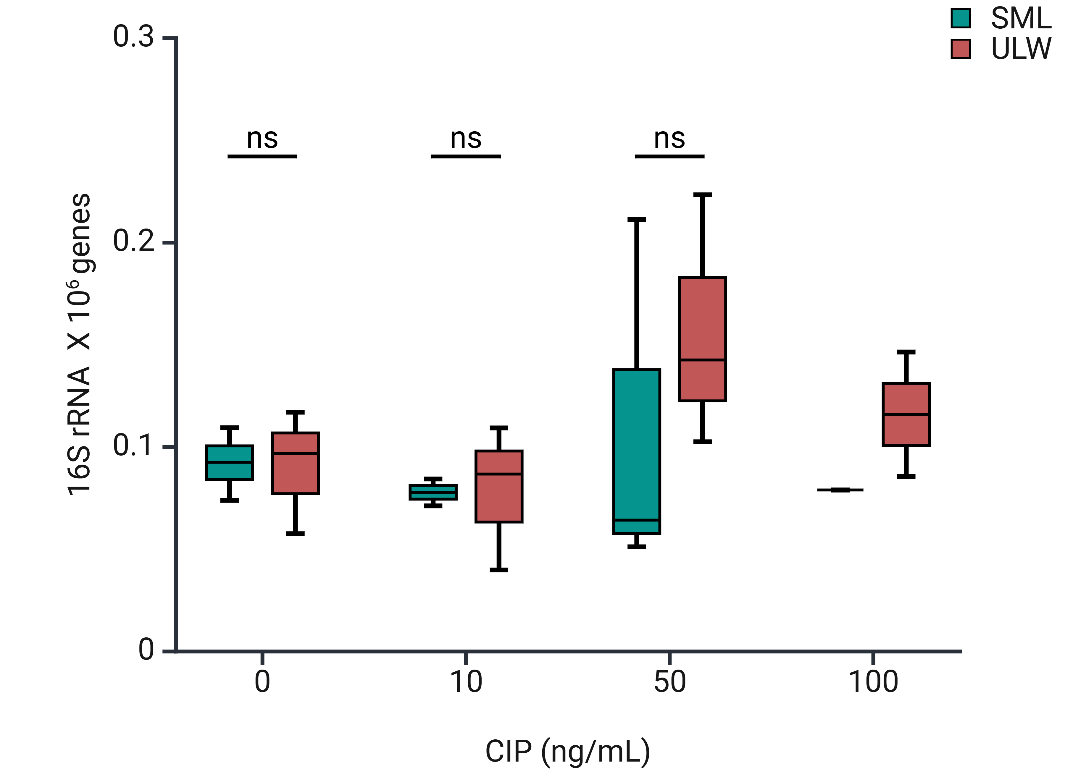


(b)

Figure S2. (a) Bacterial 16S rRNA amplicon sequence in the SML and ULW at time points. (b)16S rRNA amplicon genes in the SML and ULW samples across different ciprofloxacin concentrations. SML samples treated with 100 ng/mL at T0 and T1 were lost during sample processing. “ns" (significant difference).


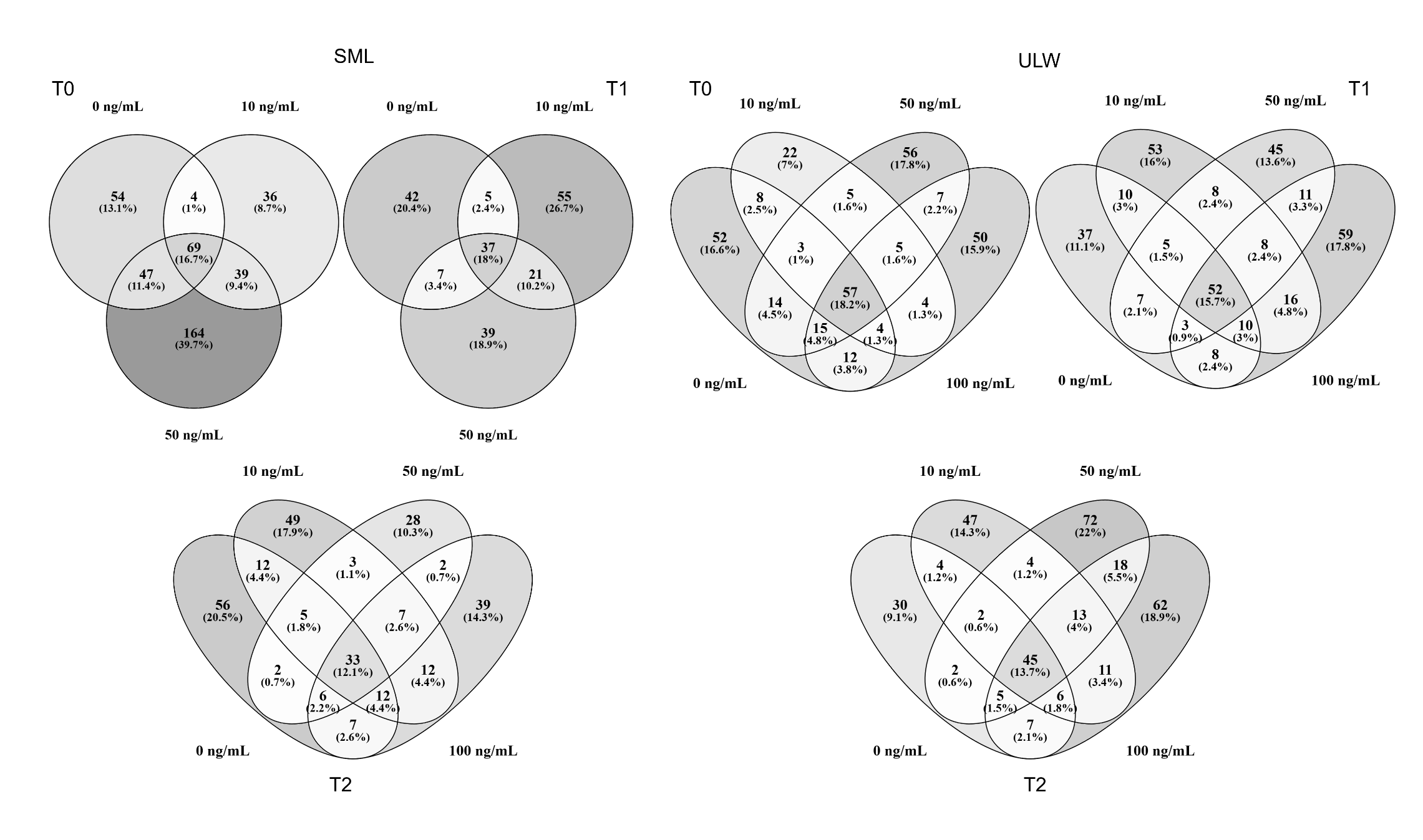
Figure S3. Venn diagrams showing individual and shared ASVs across different concentrations at different time points.

Table S1. Multiple reaction monitoring (MRM), retention time, limit of detection (LoD), and limit of quantification (LoQ) of targeted compounds (obtained from Adenaya et al. (2024))

| **Antibiotic class** | **Antibiotic type** | **MRM** | **Retention time** | **LoD**  **ng/L** | **LoQ** |
| --- | --- | --- | --- | --- | --- |
|  |  |  | **(min)** |  |  |
|  |  |  |  |  |  |
| Lincosamides | Lincomycin (+) | 407.20 > 126.00 | 2.90 | 0.02 | 0.08 |
|  | Clindamycin (+) | 425.20 > 126.00 | 4.30 | 0.02 | 0.08 |
|  | Clarithromycin (+) | 748.50 > 158.00 | 5.10 | 0.02 | 0.06 |
| Macrolides | Spiramycin (+) | 422.00 > 174.00 | 3.80 | 0.02 | 0.06 |
|  | Tylosin (+) | 916.50 > 174.00 | 4.80 | 0.02 | 0.06 |
|  | Erythromycin (+) | 734.50 > 158.00 | 4.70 | 0.02 | 0.06 |
|  | Azithromycin (+) | 748.90 > 591.00 | 5.00 | 0.01 | 0.02 |
| Quinolones | Ofloxacin (+) | 362.10 > 318.00 | 3.30 | 0.01 | 0.03 |
|  | Enrofloxacin (+) | 360.10 > 342.00 | 3.40 | 0.01 | 0.03 |
|  | Roxithromycin (+) | 837.50 > 158.00 | 5.10 | 0.00 | 0.01 |
|  | Ciprofloxacin (+) | 362.10 > 318.00 | 3.30 | 0.01 | 0.03 |
|  | Sparfloxacin (+) | 393.20 > 349.00 | 3.80 | 0.01 | 0.03 |
| Phenicol | Chloramphenicol (-) | 152.10 > 257.00 | 4.11 | 0.02 | 0.07 |
| Sulfonamides | Sulfadiazine (+) | 251.10 > 155.20 | 2.70 | 0.03 | 0.11 |
|  | Sulfadimethoxine (+) | 251.10 > 155.20 | 2.70 | 0.03 | 0.11 |
|  | Sulfamethazine (+) | 279.10 > 186.20 | 3.30 | 0.03 | 0.11 |
|  | Sulfamethizole (+) | 271.00 > 156.00 | 3.10 | 0.02 | 0.06 |
|  | Sulfamethoxazole (+) | 254.10 > 156.10 | 3.40 | 0.03 | 0.11 |
|  | Sulfaguanidine (+) | 215.10 > 156.00 | 1.63 | 0.02 | 0.06 |
|  | Sulfathiazole (+) | 256.00 > 156.00 | 2.80 | 0.02 | 0.06 |
| Aminocoumarin | Novobiocin (+) | 613.20 > 189.00 | 5.70 | 0.02 | 0.05 |
| Tetracyclines | Doxycycline (+) | 445.20 > 410.00 | 3.40 | 0.01 | 0.02 |
|  | Chlortetracycline (+) | 479.10 > 444.00 | 4.10 | 0.01 | 0.02 |
|  | Oxytetracycline (+) | 461.10 > 425.00 | 3.60 | 0.01 | 0.02 |
| Other Antibiotics | Trimethoprim (+) | 291.10 > 229.00 | 3.00 | 0.01 | 0.04 |
|  | Rifampicin (+) | 823.40 > 791.00 | 4.90 | 0.00 | 0.01 |

Table S1. ASVs' distribution across bacterial taxa

| **Taxonomic** | **Counts of** |
| --- | --- |
| **rank** | **taxonomic rank** |
| Phylum | 66 |
| Class | 161 |
| Order | 380 |
| Family | 605 |
| Genus | 1025 |
| Unknown Bacteria | 369 |

Table S3. The phylogenetic affiliation of bacterial isolates obtained on Mueller-Hinton agar (MHA) containing 100 ng/mL ciprofloxacin. Bacterial strains not identified by MALDI-TOF (not in the database) and those with MALDI-TOF scores < 2.0 were identified by sequencing their 16S rRNA genes. MALDI-TOF scores > 2.0 indicate species-level identification. Only for the isolates for which a 16S rRNA gene sequence was available, NCBI accession numbers are given. The accession numbers of the closest described relatives are also indicated for sequenced isolates.

| **Sample** | **Phylum** | **Bacterial Class/order** | **Bacterial Isolate** | **Accession** | **Closest described relative** | **Accession No** |
| --- | --- | --- | --- | --- | --- | --- |
| SML | *Bacillota* | *Bacilli/Bacillales* | *Alkalihalobacillus* sp. AN-7 |  | *Alkalihalobacillus clausii* |  |
|  |  |  | *Alkalihalobacillus* sp. AN-8 |  | *Alkalihalobacillus clausii* |  |
|  |  |  | *Bacillus* sp. AN-12 | OR739618 | *Bacillus mobilis* | MK389421.1 |
|  |  |  | *Bacillus* sp. AN-16 |  | *Bacillus coagulans* |  |
|  | *Actinomycetota* | *Actinomycetes/* | *Brevibacterium* sp. AN-14 | OR739619 | *Brevibacterium luteolum* | CP035810.1 |
|  |  | *Micrococcales* | *Brevibacterium* sp. AN-17 | OR739621 | *Brevibacterium luteolum* strain NEB1784 | CP035810.1 |
|  |  |  | *Brevibacterium* sp. AN-19 | OR739623 | *Brevibacterium luteolum* strain CF87 | NR_114872.1 |
|  |  |  | *Brevibacterium* sp. AN-20 | OR739624 | *Brevibacterium luteolum* strain NEB1784 | CP035810.1 |
|  |  |  | *Brevibacterium* sp. AN-22 | OR739626 | *Brevibacterium luteolum* strain CF87 | NR_114872.1 |
|  |  |  | Brevibacterium sp. AN-24 | OR739628 | *Brevibacterium luteolum* strain y223 | KF306374.1 |
|  |  |  | *Cytobacillus* sp. AN-21 | OR739625 | *Cytobacillus kochii* strain P140 | MZ373183.1 |
|  |  |  | *Micrococcus* sp. AN-1 |  | *Micrococcus luteus* |  |
|  |  |  | *Micrococcus* sp. AN-10 |  | *Micrococcus luteus* |  |
|  |  |  | *Micrococcus* sp. AN-11 |  | *Micrococcus luteus* |  |
|  |  |  | *Micrococcus* sp. AN-13 |  | *Micrococcus luteus* |  |
|  |  |  | *Micrococcus* sp. AN-2 |  | *Micrococcus luteus* |  |
|  |  |  | *Micrococcus* sp. AN-3 |  | *Micrococcus luteus* |  |
|  |  |  | *Micrococcus* sp. AN-6 |  | *Micrococcus luteus* |  |
|  |  |  | *Micrococcus* sp. AN-6 |  | *Micrococcus luteus* |  |
|  |  |  | *Micrococcus* sp. AN-9 |  | *Micrococcus luteus* |  |
|  | *Pseudomonadota* | *Alphaproteobacteria/* | *Ochrobactrum* sp. AN-15 | OR739620 | *Ochrobactrum chromiisoli* strain YY2X | OP787966.1 |
|  |  | *Hyphomicrobiales* | *Ochrobactrum* sp. AN-18 | OR739622 | *Ochrobactrum chromiisoli* strain YY2X | OP787966.1 |
|  |  |  | *Ochrobactrum* sp. AN-23 | OR739627 | *Ochrobactrum chromiisoli* strain YY2X | OP787966.1 |
| ULW | *Bacillota* | *Bacilli / Bacillales* | *Bacillus* sp. de-13 |  | *Bacillus cereus* |  |
|  |  |  | *Bacillus* sp. de-15 |  | *Bacillus cereus* |  |
|  |  |  | *Bacillus* sp. de-29 |  | *Bacillus cereus* |  |
|  |  |  | *Bacillus* sp. de-32 |  | *Bacillus cereus* |  |
|  |  |  | *Virgibacillus* sp. de-1 | OR739634 | *Virgibacillus proomi* | MN620396.1 |
|  |  |  | *Virgibacillus* sp. de-16 | OR739630 | *Virgibacillus proomi* | MN620396.1 |
|  |  |  | *Virgibacillus* sp. de-17 | OR739631 | *Virgibacillus proomi* | MN620396.1 |
|  |  |  | *Virgibacillus* sp. de-18 | OR739632 | *Virgibacillus proomi* | MN620396.1 |
|  |  |  | *Virgibacillus* sp. de-19 | OR739633 | *Virgibacillus proomi* | MG309540.1 |
|  |  |  | *Virgibacillus* sp. de-2 | OR739643 | *Virgibacillus proomi* | FN397532.1 |
|  |  |  | *Virgibacillus* sp. de-20 | OR739635 | *Virgibacillus proomi* | MG309540.1 |
|  |  |  | *Virgibacillus* sp. de-21 | OR739636 | *Virgibacillus proomi* | MG309540.1 |
|  |  |  | *Virgibacillus* sp. de-22 | OR739637 | *Virgibacillus proomi* | MN620396.1 |
|  |  |  | *Virgibacillus* sp. de-24 | OR739638 | *Virgibacillus proomi* | MG309540.1 |
|  |  |  | *Virgibacillus* sp. de-25 | OR739639 | *Virgibacillus proomi* | MG309540.1 |
|  |  |  | *Virgibacillus* sp. de-26 | OR739640 | *Virgibacillus proomi* | MG309540.1 |
|  |  |  | *Virgibacillus* sp. de-27 | OR739641 | *Virgibacillus proomi* | MG309540.1 |
|  |  |  | *Virgibacillus* sp. de-28 | OR739642 | *Virgibacillus proomi* | MG309540.1 |
|  |  |  | *Virgibacillus* sp. de-3 | OR739645 | *Virgibacillus proomi* | MN620396.1 |
|  |  |  | *Virgibacillus* sp. de-30 | OR739644 | *Virgibacillus proomi* |  |
|  |  |  | *Virgibacillus* sp. de-7 |  | *Virgibacillus proomi* |  |
|  |  |  | *Virgibacillus* sp. de-9 |  | *Virgibacillus proomi* |  |
|  | *Actinomycetota* | *Actinomycetes/* | *Micrococcus* sp. de-10 |  | *Micrococcus luteus* |  |
|  |  | *Micrococcales* | *Micrococcus* sp. de-11 |  | *Micrococcus luteus* |  |
|  |  |  | *Micrococcus* sp. de-12 |  | *Micrococcus luteus* |  |
|  |  |  | *Micrococcus* sp. de-14 |  | *Micrococcus luteu* |  |
|  |  |  | *Micrococcus* sp. de-23 |  | *Micrococcus luteus* |  |
|  |  |  | *Micrococcus* sp. de-31 |  | *Micrococcus luteus* |  |
|  |  |  | *Micrococcus* sp. de-4 |  | *Micrococcus luteus* |  |
|  |  |  | *Micrococcus* sp. de-5 |  | *Micrococcus luteus* |  |
|  |  |  | *Micrococcus* sp. de-6 |  | *Micrococcus luteus* |  |
|  |  |  | *Micrococcus* sp. de-8 |  | *Micrococcus luteus* |  |
